# The novel interaction between *Neisseria gonorrhoeae* TdfJ and human S100A7 allows gonococci to subvert host zinc restriction

**DOI:** 10.1371/journal.ppat.1007937

**Published:** 2019-08-01

**Authors:** Stavros Maurakis, Kayla Keller, C. Noel Maxwell, Kevin Pereira, Walter J. Chazin, Alison K. Criss, Cynthia Nau Cornelissen

**Affiliations:** 1 Department of Microbiology and Immunology, Virginia Commonwealth University School of Medicine, Richmond, VA, United States of America; 2 Biomedical Sciences Doctoral Portal, Virginia Commonwealth University School of Medicine, Richmond, VA, United States of America; 3 Departments of Biochemistry and Chemistry, Vanderbilt University, Nashville, Tennessee, United States of America; 4 Department of Microbiology, Immunology, and Cancer Biology, University of Virginia, Charlottesville, VA, United States of America; 5 Institute for Biomedical Sciences, Georgia State University, Atlanta, GA, United States of America; University of Oxford, UNITED KINGDOM

## Abstract

*Neisseria gonorrhoeae* causes the sexually-transmitted infection gonorrhea, a global disease that is difficult to treat and for which there is no vaccine. This pathogen employs an arsenal of conserved outer membrane proteins called TonB-dependent transporters (TdTs) that allow the gonococcus to overcome nutritional immunity, the host strategy of sequestering essential nutrients away from invading bacteria to handicap infectious ability. *N*. *gonorrhoeae* produces eight known TdTs, of which four are utilized for acquisition of iron or iron chelates from host-derived proteins or xenosiderophores produced by other bacteria. Of the remaining TdTs, two of them, TdfH and TdfJ, facilitate zinc uptake. TdfH was recently shown to bind Calprotectin, a member of the S100 protein family, and subsequently extract its zinc, which is then internalized by *N*. *gonorrhoeae*. Like Calprotectin, other S100s are also capable of binding transition metals such as zinc and copper, and thus have demonstrated growth suppression of numerous other pathogens via metal sequestration. Considering the functional and structural similarities of the TdTs and of the S100s, as well as the upregulation in response to Zn limitation shown by TdfH and TdfJ, we sought to evaluate whether other S100s have the ability to support gonococcal growth by means of zinc acquisition and to frame this growth in the context of the TdTs. We found that both S100A7 and S10012 are utilized by *N*. *gonorrhoeae* as a zinc source in a mechanism that depends on the zinc transport system ZnuABC. Moreover, TdfJ binds directly to S100A7, from which it internalizes zinc. This interaction is restricted to the human version of S100A7, and zinc presence in S100A7 is required to fully support gonococcal growth. These studies highlight how gonococci co-opt human nutritional immunity, by presenting a novel interaction between TdfJ and human S100A7 for overcoming host zinc restriction.

## Author summary

*Neisseria gonorrhoeae* causes the common sexually-transmitted infection gonorrhea. This bacteria’s ability to rapidly acquire antibiotic resistance factors, coupled with the lack of any effective vaccine to prevent infection, has resulted in a disease that poses a global threat and may become untreatable. A group of gonococcal outer membrane proteins called TonB-dependent transporters (TdTs) have been implicated as promising vaccine targets, as they are well-conserved and expressed across gonococcal isolates and play a vital role in allowing the pathogen to acquire essential nutrients during infection of the human host. Here, we describe the conservation and regulation of TdfJ, a gonococcal TdT whose homologues are ubiquitous in the genus *Neisseria*. We show that TdfJ binds directly to S100A7, a host protein that normally sequesters zinc away from invading pathogens. This novel interaction enables *N*. *gonorrhoeae* to strip S100A7 of chelated zinc for its own use. Furthermore, we show that another zinc-binding human protein, S100A12, is also utilized by *N*. *gonorrhoeae* as a zinc source by an as-yet-unidentified mechanism. This study provides insight into the functional role of the TdTs during infection and highlights these proteins as promising targets for both vaccine and antimicrobial therapy development.

## Introduction

The obligate human pathogen *Neisseria gonorrhoeae*, the etiological agent of the eponymous sexually-transmitted infection (STI) gonorrhea, poses a growing threat to global health. In 2017, the World Health Organization (WHO) estimated over 100 million new cases of gonorrhea, with surveillance from the Centers for Disease Control and Prevention (CDC) estimating 555,000 new, reported cases of this disease each year in the United States alone, and an increase in cases of 18.6% from 2016–2017 [1]. Gonorrhea afflicts both men and women, with symptomatic infections in men presenting as urethritis and epididymitis, and in women as cervicitis. Concerningly, up to 80% of cases in women can be asymptomatic [2], leading to potential ascension into the upper reproductive tract and manifestations including disseminated infection, pelvic inflammatory disease and in some cases even infertility and ectopic pregnancy. Moreover, gonococcal infection poses a serious economic burden, with yearly estimates for treatment of gonorrhea in the United States reaching as high as $240 million [3].

The window of available treatment options for infection by *N*. *gonorrhoeae* is rapidly narrowing, as this naturally-competent pathogen rapidly acquires and stably maintains factors for antimicrobial drug resistance. The current treatment regimen for gonorrhea recommended by the CDC is a dual therapy of ceftriaxone and azithromycin [4], as resistance to quinolones, penicillins, and sulfonamides is ubiquitous, and recent reports have shown treatment failures when using extended-spectrum cephalosporins alone [5–7]. Critically, gonococcal isolates resistant to all forms of approved therapy including the recommended dual therapy have been identified, thus creating a legitimate threat that gonorrhea may become untreatable, and highlighting the need for research into novel means of treatment and/or prevention of gonococcal disease [8]. In 2017, the WHO placed *N*. *gonorrhoeae* on its watch list as a high priority pathogen for which new treatment strategies are needed.

No effective vaccine for *N*. *gonorrhoeae* currently exists, and infection by the gonococcus does not confer any protective immunity against subsequent infections [9, 10]. High-frequency phase and antigenic variation in *N*. *gonorrhoeae* result in a bacterial surface that is constantly variable, with certain structures changing at rates up to 10^−2^ cells in a single population [11]. The net result of this variation is that the pathogen evades both the host’s natural adaptive immunity and presents a limited number of promising targets for vaccine development [12]. Identification of these targets is paramount.

**Fig 1:**
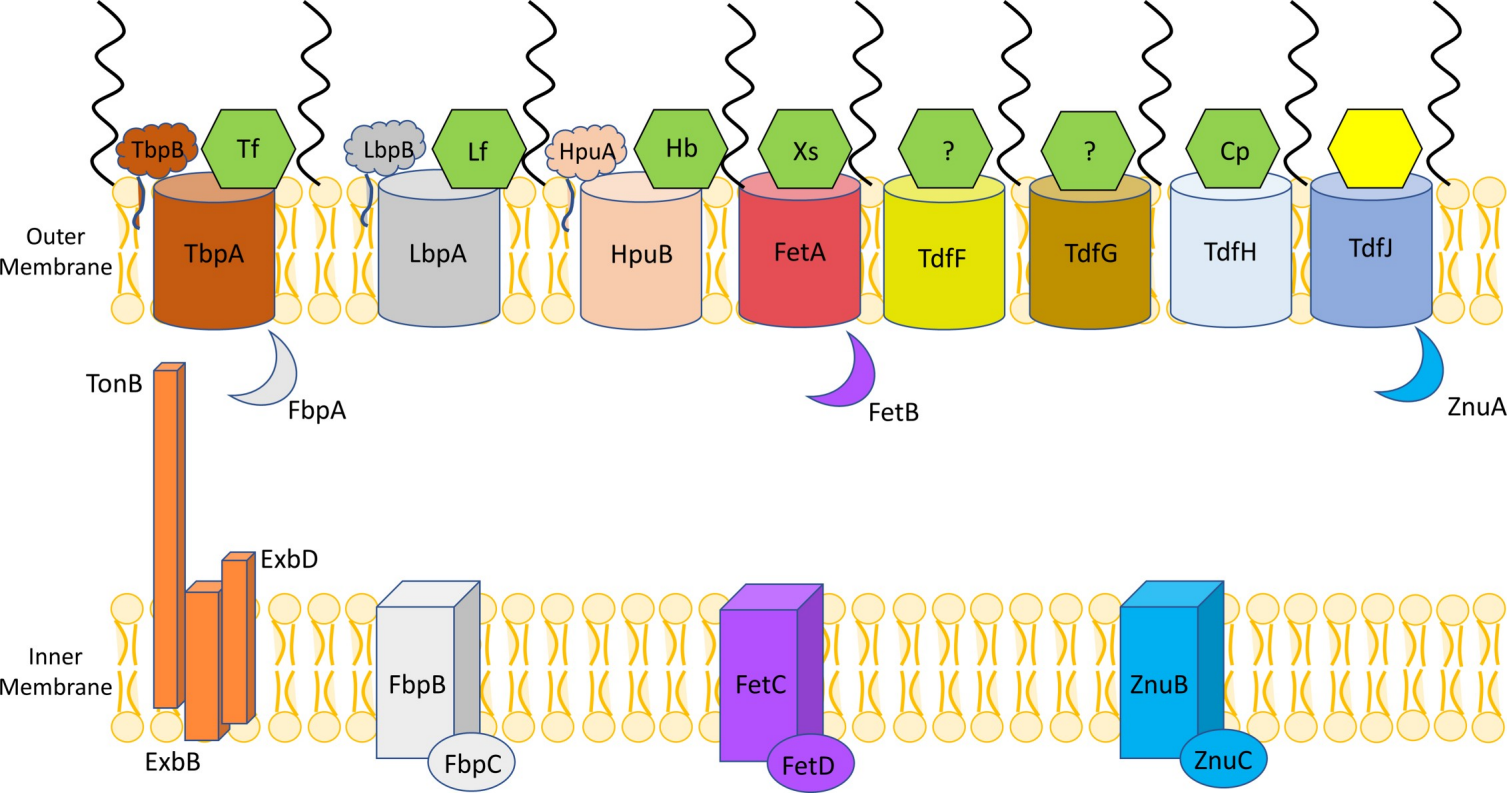
TonB-dependent transporters, surface-tethered lipoproteins, and ABC transport systems produced by *Neisseria gonorrhoeae*. Cartoon schematics of the eight known TdTs produced by the gonococcus are represented by barrel shapes in the outer membrane, and their respective ligands identified to date, shown by green hexagons. The ligand for TdfJ, which is the focus of this manuscript, is highlighted in yellow. Also illustrated are the lipoprotein components associated with TbpA, LbpA, and HpuB, shown here anchored to the outer membrane. TonB, ExbB, and ExbD, which utilize the proton motive force to energize the transport of cargo through the outer membrane via the TdTs are shown in the left-hand side of the inner membrane. Black squiggles on the outer leaflet represent lipooligosaccharide. Lastly, the cognate ABC transport systems associated with the TdTs are shown in the inner membrane and the periplasmic space. FbpABC transports ferric iron, FetBCD transports catecholate-type siderophores, and ZnuABC (also called MntABC elsewhere in the literature) transports zinc and manganese. Ligands: Tf = Transferrin; Lf = Lactoferrin; Hb = Hemoglobin; Xs = Xenosiderophore; Cp = Calprotectin.

A promising set of surface structure targets are the highly-conserved TonB-dependent transporters (TdTs), which play a vital role in acquisition of nutrients for the gonococcus [13]. The TdTs are expressed across the genus *Neisseria* and show limited sequence variation. These outer membrane proteins consist of a β-barrel domain that is occluded by a globular plug, and feature extracellular loops that interact with nearby ligands. Upon binding the correct ligand, transport of specific cargo (in this context, nutrients) is stimulated by the TonB protein. TonB, which is situated in the inner membrane in complex with two other proteins, ExbB and ExbD, utilizes the proton motive force to energize passage of cargo though the β-barrel by modulating conformational change in the plug domain [14, 15]. Once the cargo has reached the periplasm, it is taken across the cytoplasmic membrane by a dedicated ABC transport system. *N*. *gonorrhoeae* produces eight known TdTs, and those that have been characterized play a key role in acquisition of transition metals such as iron and zinc [13]. *Neisserial* TdTs have been demonstrated to interact with host-derived proteins including transferrin, lactoferrin, and hemoglobin for iron uptake [16], and more recent studies have revealed the S100 protein Calprotectin as a zinc-binding TdT ligand. Stork et al. showed that the meningococcal protein CbpA allows utilization of Calprotectin as a zinc source [17], and our lab recently demonstrated that the gonococcal homologue of this protein, called TdfH, is also capable of binding human Calprotectin and stripping it of zinc, which is then accumulated within the gonococcus [18]. An overview of these TdTs and their accessory proteins and known ligands is shown in Fig 1. The process of metal piracy is implicated as a critical virulence factor for *N*. *gonorrhoeae*, as a human male infection model demonstrated that mutant gonococci incapable of utilizing iron from transferrin and lactoferrin were incapable of infection [19], and TdfH production allows the pathogen to survive in Neutrophil Extracellular Traps (NETs), which are highly enriched for Calprotectin, *in vitro* [18]. Gonococcal circumvention of killing by NETs is particularly nefarious, as *N*. *gonorrhoeae* specifically elicits a robust neutrophil response during infection [20, 21], thus effectively tricking the host into bringing a vital nutrient directly to the invading pathogen.

Considered together, the TdTs discussed are part of the arsenal deployed by *N*. *gonorrhoeae* to overcome nutritional immunity [16]. Nutritional immunity refers to the host strategy of sequestering essential nutrients in order to restrict their availability to invading pathogens and therefore handicap their growth and infection potential. This phenomenon has been described in detail for iron, zinc, and manganese [22–27]. For bacteria, approximately 6% of proteins require zinc as either a key structural or catalytic component. Of these 6%, approximately 80% are enzymatic in nature, and zinc structural sites such as zinc fingers are common [28], making zinc a necessity for bacterial survival. The human host has evolved nutritional immunity mechanisms specific for zinc sequestration. The previously mentioned protein Calprotectin binds to zinc with high affinity, and accounts for over 40% of the cytosolic protein in neutrophils [29]. Although this protein has proven incapable of inhibiting growth of *N*. *gonorrhoeae* [18], it has demonstrated innate immune functions against other pathogens including *Escherichia coli*, *Staphylococcus aureus*, and *Candida albicans* [30–36] due to its ability to chelate zinc and manganese. Calprotectin is a member of the S100 calcium binding protein family, of which there are currently 24 identified members divided according to their primary location of function at the cellular level: intracellular only, extracellular only, or both [37]. They are differentially expressed according to tissue type. The S100s are EF-hand proteins whose primary role in vertebrates is modulation of calcium homeostasis, though they participate in other processes including inflammation, differentiation, apoptosis, and regulation of polymorphonuclear cells [37]. S100 proteins are obligate dimers and also form higher order oligomers, and are able to bind transition metals via sites at the dimer interface including zinc, manganese, copper, nickel and iron [24, 38, 39]. As such, several S100 proteins, in addition to Calprotectin, have demonstrated suppressive effects on bacterial growth through sequestration of these metals. S100A7, also called Psoriasin due to its upregulation in psoriatic lesions [40, 41], has demonstrated zinc-mediated antimicrobial activity against *S*. *aureus*, *Pseudomonas aeruginosa*, *and E*. *coli* [42–47]. S100A12 (Calgranulin C) has demonstrated similar effects against several bacteria and parasites [42, 48–50]. These findings highlight the importance of zinc at the host-pathogen interface and emphasize the need for further investigation into pathogen strategies to circumvent host nutrient depravation.

Herein, we describe the conservation, regulation, and phenotypic characterization of another TdT produced by *N*. *gonorrhoeae*: TdfJ. TdfJ has been described as having differential responses to the presence of iron and zinc [18], and both TdfJ and its meningococcal homologue, ZnuD, have demonstrated a contribution to growth of *Neisseria* in zinc-deplete conditions [51]. Furthermore, ZnuD directly binds to zinc *in vitro* [52]. In the current study, we demonstrate that gonococcal TdfJ shows additive repression by zinc and induction by iron. Furthermore, we show that wild-type *N*. *gonorrhoeae* is capable of utilizing zinc bound to S100A7 and S100A12 as a sole zinc source, and that utilization of S100A7 depends on the function of TdfJ, TonB, and the associated ABC transporter ZnuABC [53]. We demonstrate that S100A7 binds directly to both gonococcal and recombinant TdfJ in whole-cell solid-phase binding assays, and show via inductively coupled plasma optical emission spectrometry (ICP-OES) that the interaction between these two proteins allows *N*. *gonorrhoeae* to accumulate zinc internally. Finally, we demonstrate that TdfJ’s interaction with S100A7 is restricted to the human version of the protein, and that growth support by S100A7 depends on its molecular nature as a zinc chelator. This report marks the first time that S100A7 has been posited to be bound and directly utilized as a zinc source by an invading pathogen.

## Results

### The *tdfJ* gene is found in all *Neisseria* species and is highly conserved

**Fig 2:**
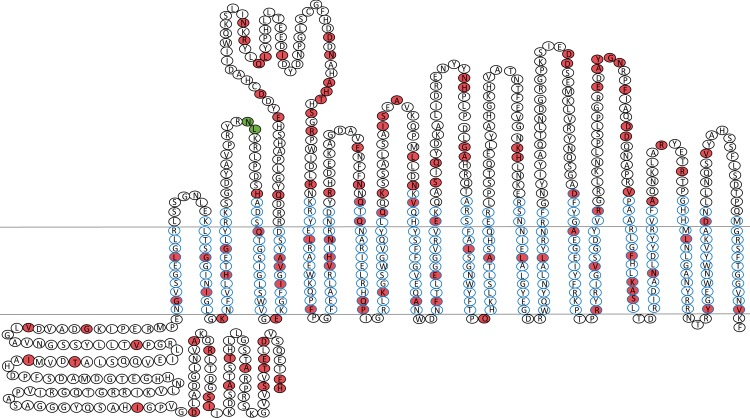
Predicted topology and conservation of TdfJ and its homologues. Amino acid sequences for 50 examples of gonococcal TdfJ or its homologue in *N*. *meningitidis*, *N*. *lactamica*, or *N*. *elongata* (accession numbers are available in the Materials and Methods) were aligned using Geneious and submitted to NetSurfP-2.0 for prediction of transmembrane domains and intra-/extracellular loops. This information was used to create a topology prediction in TOPO2, which was then adapted to create the above model, which has the protein’s cleavable N-terminal signal sequence removed. The two horizontal lines represent the outer membrane, and each amino acid residue of the protein is represented by a circle. Residues in white are identical across all 50 sequences, while those in red have 1 or more mismatches, and the most common residue is indicated. Residues outlined in blue make up β sheets. Two residues are shown in green. These two residues are variable, and 9 of the 50 sequences queried contain a conserved KEEG motif between these two residues. The sequences tested showed 96.2% pairwise identity, with 82.9% of residues being identical in all cases.

In order to assess TdfJ as a vaccine candidate, we evaluated the predicted amino acid sequence identity among the TdfJ proteins or their homologues in *Neisseria* genomes available in NCBI databases, and found that the protein is highly identical across all queried isolates. To demonstrate this, we performed multiple alignment of 50 of these sequences in Geneious (Biomatters, Ltd.) and found that the proteins shared 96.2% pairwise identity. Furthermore, 82.9% of amino acid residues in the proteins were identical across all sequences. It should be noted that a few sequences selected contain a six-residue stretch (MRREAK) at the amino terminus, as there is some database inconsistency as to which of two close-proximity methionines is the true start of the protein. For the purposes of alignment, these residues were ignored, but all six are identical in the sequences which include them. We also generated a consensus sequence from those queried and submitted it to DTU Bioinformatics NetSurfP-2.0 (http://www.cbs.dtu.dk/services/NetSurfP/) for domain prediction, which identified putative β strands that constitute the protein’s transmembrane domains. Using the consensus sequence and predicted β barrel, we generated a topology prediction for TdfJ using TOPO2 from the University of California at San Francisco (http://www.sacs.ucsf.edu/cgi-bin/open-topo2.py). The resulting model was adapted to show the conserved and variable residues of TdfJ within the 2D topology diagram (Fig 2). The meningococcal homologue of TdfJ, ZnuD, has been previously crystallized and shows hallmark β barrel and globular plug domains characteristic of the TonB-dependent transporters [52]. Although gonococcal TdfJ has not yet been crystallized, based on these data we predict it to be virtually identical in structure to ZnuD, which has received considerable attention as a potential vaccine candidate for *N*. *meningitidis* [51, 54]. For these reasons we sought to further investigate TdfJ and its potential contributions to the advancement of gonococcal vaccine development.

### Gonococcal TdfJ is zinc repressed and iron induced

**Fig 3:**
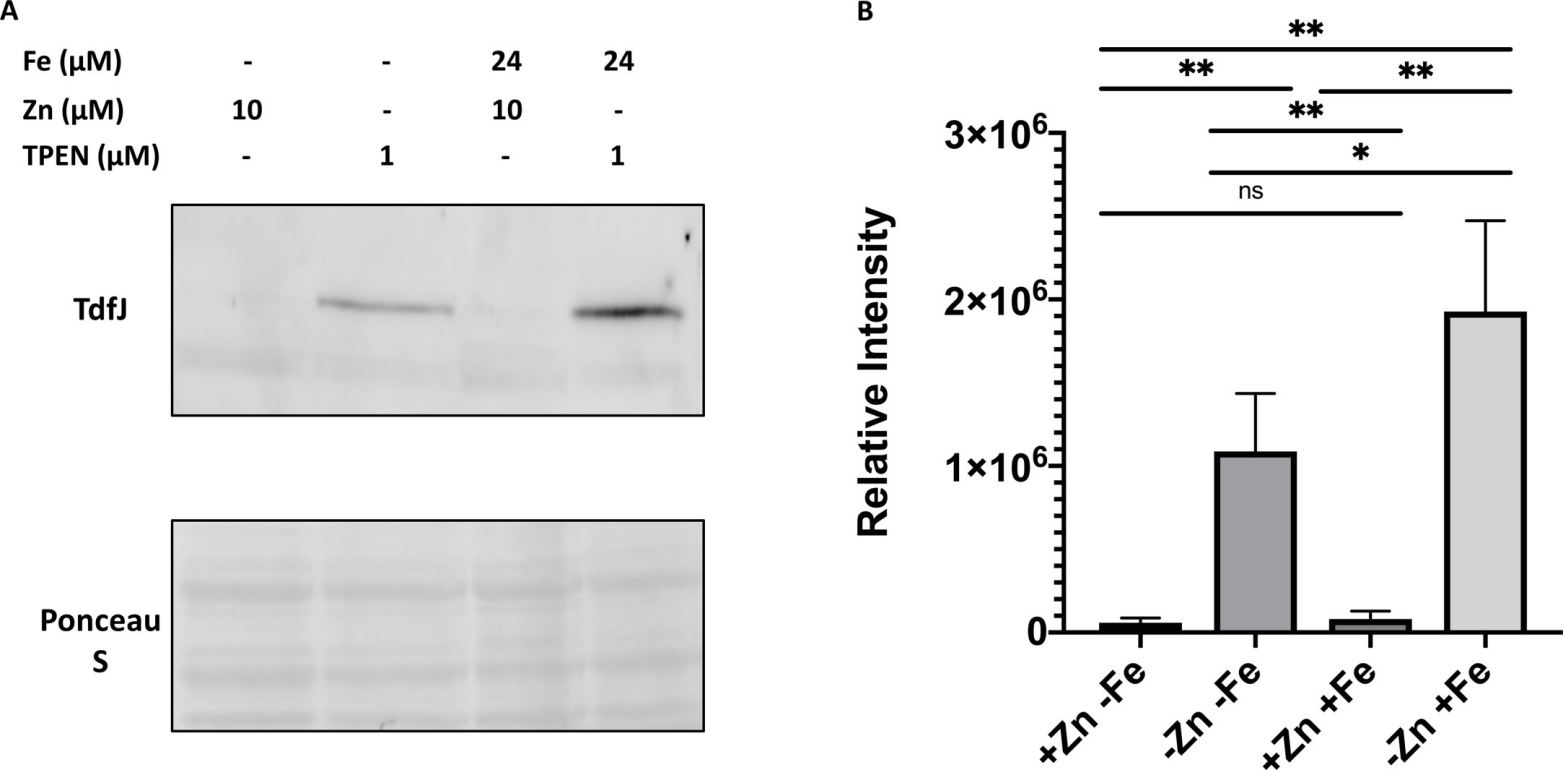
TdfJ is zinc repressed and iron induced. (A) Strain FA19 was grown in CDM until exponential growth, then back diluted in the same media and treated with Fe(NO_3_)_3_, ZnSO_4_, and/or TPEN in the concentrations indicated. These cultures were grown for 6 hours before whole-cell lysates of equalized density were prepared and subjected to SDS-PAGE and subsequent immunoblotting with antiserum raised against TdfJ. The Ponceau stained blot shows equal protein loading. Blot is representative of N = 4 experiments. (B) Western blots shown in panel (A) were analyzed for signal intensity using BioRad Image Lab software. Statistical significance was determined using an unpaired Welch’s t-test. *, P ≤. 05; **, P ≤ .005; ***, P ≤ .0005; ****, P ≤ .0001.

We next identified the conditions under which TdfJ was maximally produced in *N*. *gonorrhoeae* wild-type strain FA19 [55]. We previously reported that strain FA1090 shows decreased TdfJ production in the presence of zinc and increased production in the presence of iron [18], but the additive effects of these two metals in strain FA19 were not assessed. We performed western analysis of whole-cell lysates prepared from strain FA19 grown in Chelex-treated chemically defined media (CDM) supplemented with zinc, iron, and/or the high affinity zinc chelator *N*,*N*,*N′*,*N′*-tetrakis-(2-pyridinylmethyl)-1,2-ethanediamine (TPEN) at the concentrations indicated. We found that levels of TdfJ were decreased in the presence of zinc and increased in zinc-deplete conditions (Fig 3A). TdfJ production was almost nonexistent when zinc was present and iron absent, which agrees with the observed phenotype for FA1090 [18]. When both zinc-deplete and iron-replete conditions were established, we observed an additive effect of their respective regulation inputs. Equal sample loading was verified by Ponceau stain of the protein blot. We then performed densitometry analysis on N = 3 blots and verified that zinc-restricted bacteria produce significantly more TdfJ than those grown in zinc-replete conditions, and that when zinc is scarce, TdfJ production is significantly higher when iron is present than when it is absent (Fig 3B). These results established for us a set of conditions that are optimal for TdfJ production, and these were kept consistent in subsequent experiments.

### Wild-type gonococci can utilize zinc bound to S100A7 and S100A12 *in vitro*

**Fig 4:**
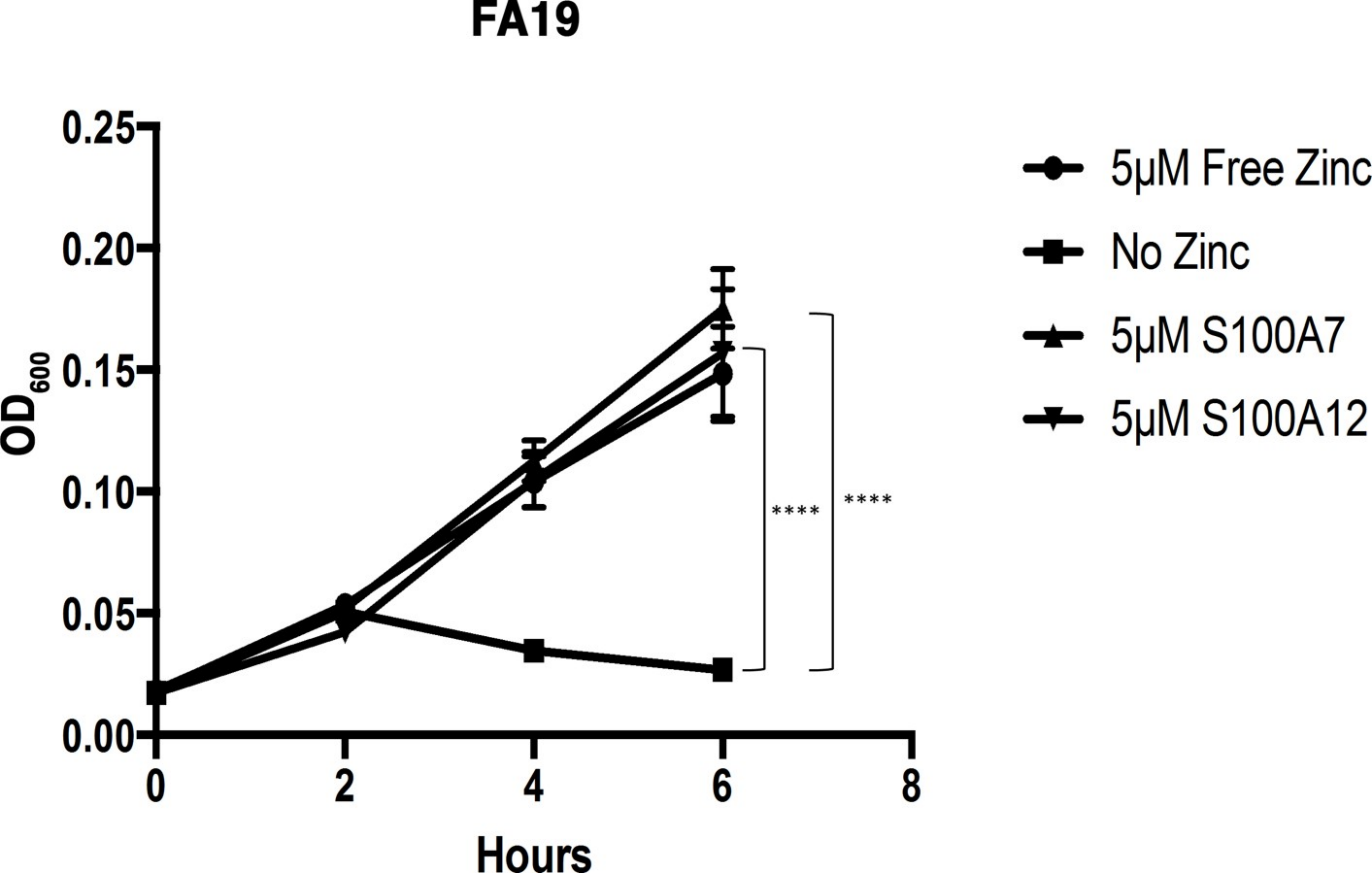
*Neisseria gonorrhoeae* strain FA19 utilizes S100A7 and S100A12 as a sole zinc source. Wild-type *N*. *gonorrhoeae* strain FA19 was grown in Zn-limited conditions in defined media (CDM), which was supplemented with growth premix containing either ZnSO_4_, S100A7, S100A12, or no zinc. Growth was measured by OD_600_ readings every 2 h for 6 h total. The graph demonstrates means and SD for N = 3 independent experiments. A 2-way repeated measures ANOVA with Tukey’s correction was performed for all means, with significance at 6 h shown. *, P ≤. 05; **, P ≤ .005; ***, P ≤ .0005; ****, P ≤ .0001.

Considering the regulation of TdfJ by zinc, we hypothesized that TdfJ contributes to zinc acquisition by the gonococcus, and that the zinc-bearing ligand for TdfJ belongs to the S100 family of proteins. To test this hypothesis, we assayed the ability of wild-type strain FA19 to grow in the presence of S100 proteins that are reported to bind zinc. For these studies the dimeric S100 proteins were loaded to ~25% saturation with zinc in zinc-deplete conditions. Strikingly, we found that S100A7 and S100A12, which have antimicrobial activity against other pathogens [42], were able to support growth of the gonococcus as a sole zinc source in a manner similar to that observed in the presence of ZnSO_4_ alone (Fig 4). When no zinc source was provided, the gonococci ceased to grow after approximately 2 hours. We hypothesize that the bacteria’s internal zinc stores could sustain it for roughly one doubling before no longer being sufficient. These data imply that *N*. *gonorrhoeae* is able to overcome host nutritional immunity by directly co-opting host zinc-sequestering proteins S100A7 and S100A12, similar to Calprotectin as we previously observed [18].

**Fig 5:**
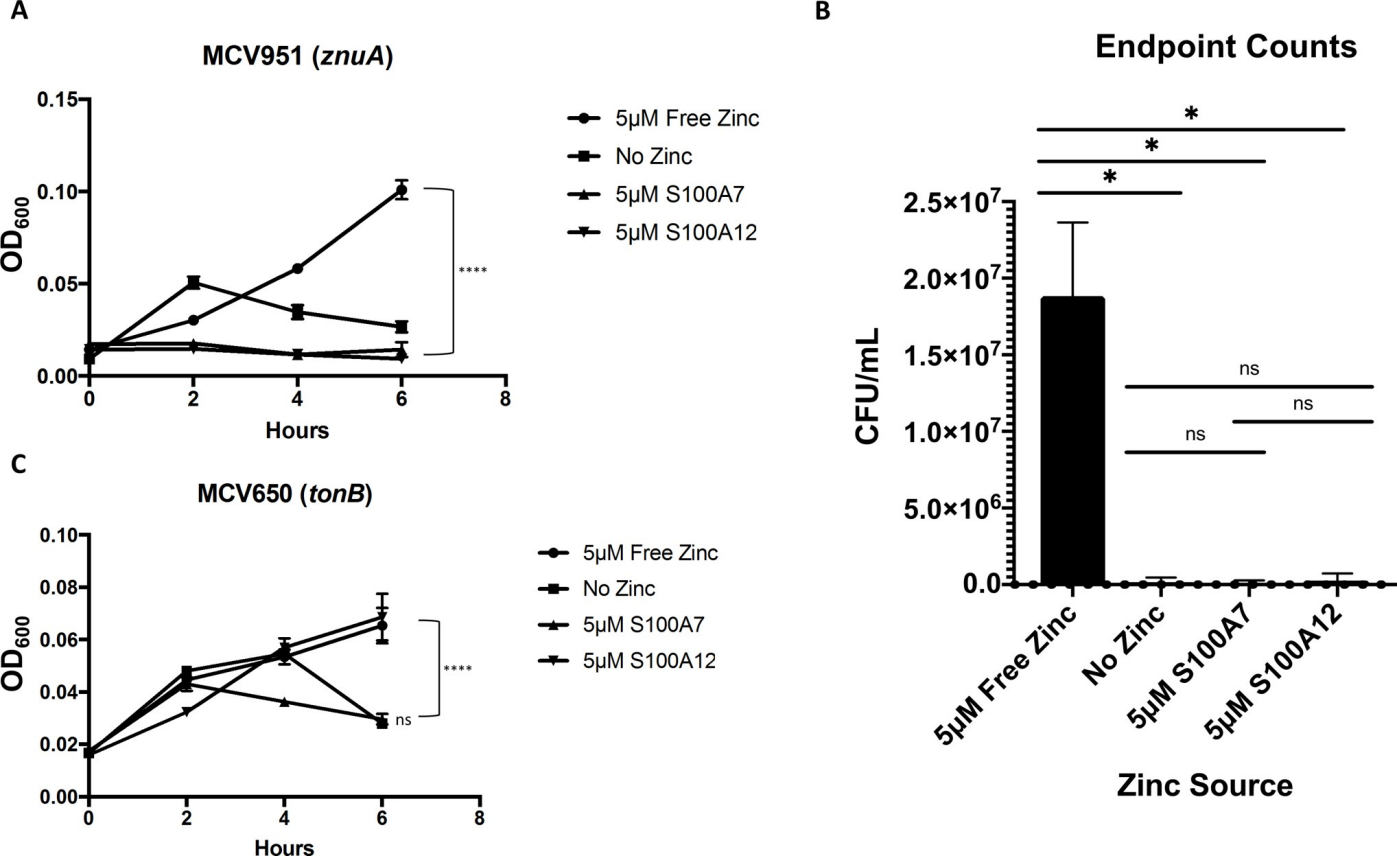
S100A7 utilization during growth depends on ZnuA and TonB. (A) *N*. *gonorrhoeae* mutant strain MCV951 (*znuA*) was grown in Zn-limited conditions in defined media (CDM), which was supplemented with growth premix containing either ZnSO_4_, S100A7, S100A12, or no zinc. Growth was measured by OD_600_ readings every 2 h for 6 h total. (B) The *znuA* mutant strain, MCV951, was submitted to liquid growth assays to assess its ability to utilize free zinc and zinc bound to either S100A7 or S100A12. After 6 hours of assay time, samples grown with each zinc source were collected, serially diluted in PBS, and spotted onto GCB plates supplemented with 5 mM D-Mannitol. After incubation for 24 h, colonies were enumerated to demonstrate that this strain does indeed grow in the presence of useable zinc despite the inability to produce the ZnuA protein. Significance was calculated using an unpaired Welch’s T-test performed on N = 3 assay sets. (C) *N*. *gonorrhoeae* mutant strain MCV650 (*tonB*) was also grown in Zn-restricted CDM, but to overcome the mutant’s inability to utilize iron from human transferrin, growth premix was instead prepared with 3 μM Fe(NO_3_)_3_ with iron chelators excluded. Both graphs demonstrate means and SD for N = 3 independent experiments. A 2-way repeated measures ANOVA with Tukey’s correction was performed for all means, with significance at 6 h shown. *, P ≤. 05; **, P ≤ .005; ***, P ≤ .0005; ****, P ≤ .0001.

**Fig 6:**
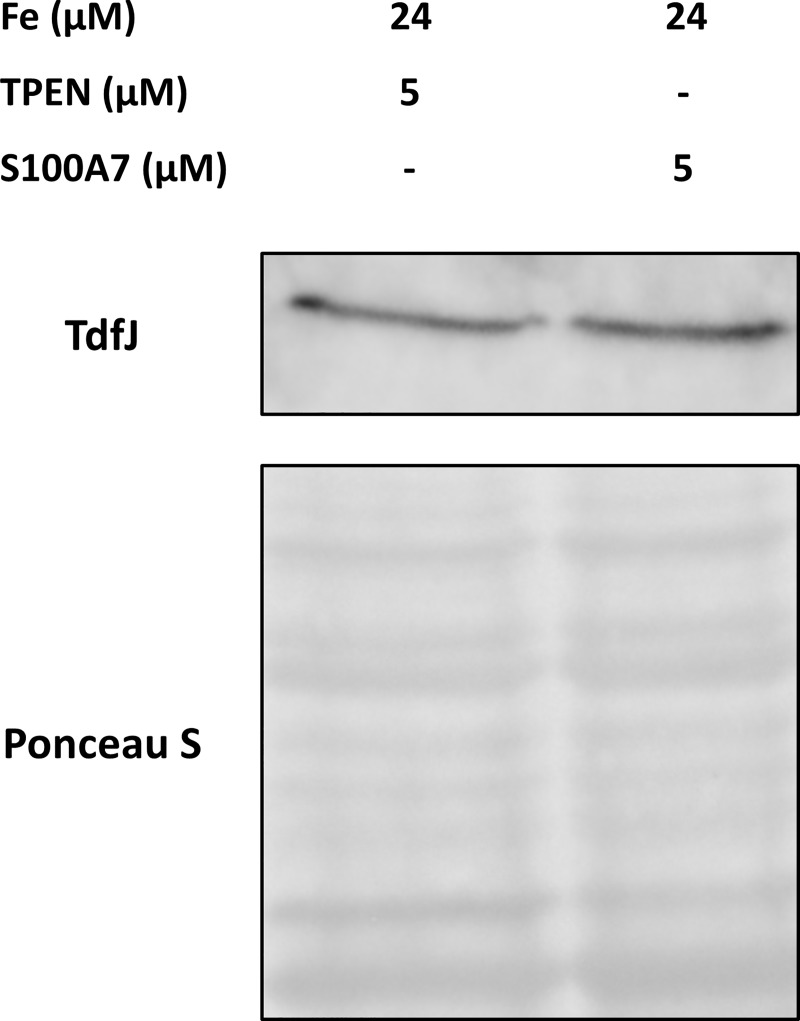
S100A7 presence induces production of TdfJ. Strain FA19 was grown in CDM until exponential growth, then back diluted in the same media and treated with Fe(NO_3_)_3_, and either TPEN or S100A7 in the concentrations indicated. These cultures were grown for 6 hours before whole-cell lysates of equalized density were prepared and subjected to SDS-PAGE and western blotting to detect TdfJ. Equal protein loading is demonstrated by Ponceau staining. N = 3 represented.

To elucidate the contribution of the Tdfs in gonococcal utilization of zinc-binding S100 proteins, we first constructed a mutant strain in the FA19 background that was incapable of producing ZnuA, the periplasmic binding portion of the ABC transport system for zinc [53]. This mutant, MCV951, was unable to grow when presented with S100A7 or S100A12 as a sole zinc source and grew only poorly when supplemented with free ZnSO_4_ (Fig 5A). This supports the hypothesis that ZnuABC participates in zinc uptake, and is also in line with previous observations that gonococci defective for this ABC transport system show a severe growth defect due to ZnuABC’s role in managing oxidative stress. Gonococcal ZnuABC has elsewhere been given the name MntABC due to its ability to bind and transport manganese [56–58]. With the understanding that *znuA* mutant gonococci are considerably hindered in their growth capabilities, we conducted endpoint viable counts of all samples in this growth assay to confirm that bacteria were able to survive when presented with a useable zinc source. These experiments indicated that at the 6-hour timepoint, the MCV951 strain was still viable when grown in the presence of free zinc (Fig 5B), which presumably entered the cells in small amounts via passive diffusion. The first result that distinguished between S100A7 and S100A12 came in their respective abilities to support growth of an isogenic *tonB* mutant, MCV650 [59] (Fig 5C). While S100A12 supported growth in a manner similar to that of free ZnSO_4_ and was not impacted by the *tonB* mutation, samples containing S100A7 grew no better than those without zinc. The inability of the gonococcus to utilize S100A7 in the absence of either ZnuA or TonB suggests that the interaction between pathogen and ligand may be mediated by one of the Tdfs. It is important to note that MCV650 cannot use the preferred iron source in our assay, which is iron-saturated human transferrin, as TonB is required for this process [60]. Instead, these samples were provided with iron in the form of free Fe(NO_3_)_3_, which is not utilized as efficiently by the gonococcus. This may explain the overall poor growth and unusual shape of the growth curves for MCV650. To ensure that the iron source present did not have any effect on zinc-dependent growth, growth experiments were performed with both Fe(NO_3_)_3_ and human transferrin. Overall growth was lower for samples containing Fe(NO_3_)_3_, but all trends were recapitulated in both sets of samples. Considering the fact that utilization of S100A7 depends on function of the TonB protein, we analyzed whether its presence altered the expression of TdfJ, as this would be the expected outcome for a zinc-sequestering protein affecting a zinc-responsive gene. To test this, we grew strain FA19 in CDM supplemented with Fe(NO_3_)_3_ and equal amounts of either TPEN or apo-S100A7 to induce zinc stress. Western analysis of these samples indicated that zinc stress imposed by S100A7 results in TdfJ protein levels that are indistinguishable from those induced by TPEN presence (Fig 6). Collectively, these data suggest that TdfJ may be associated with S100A7 utilization.

### TdfJ is necessary for gonococci to bind S100A7 and utilize it as a sole zinc source

**Fig 7:**
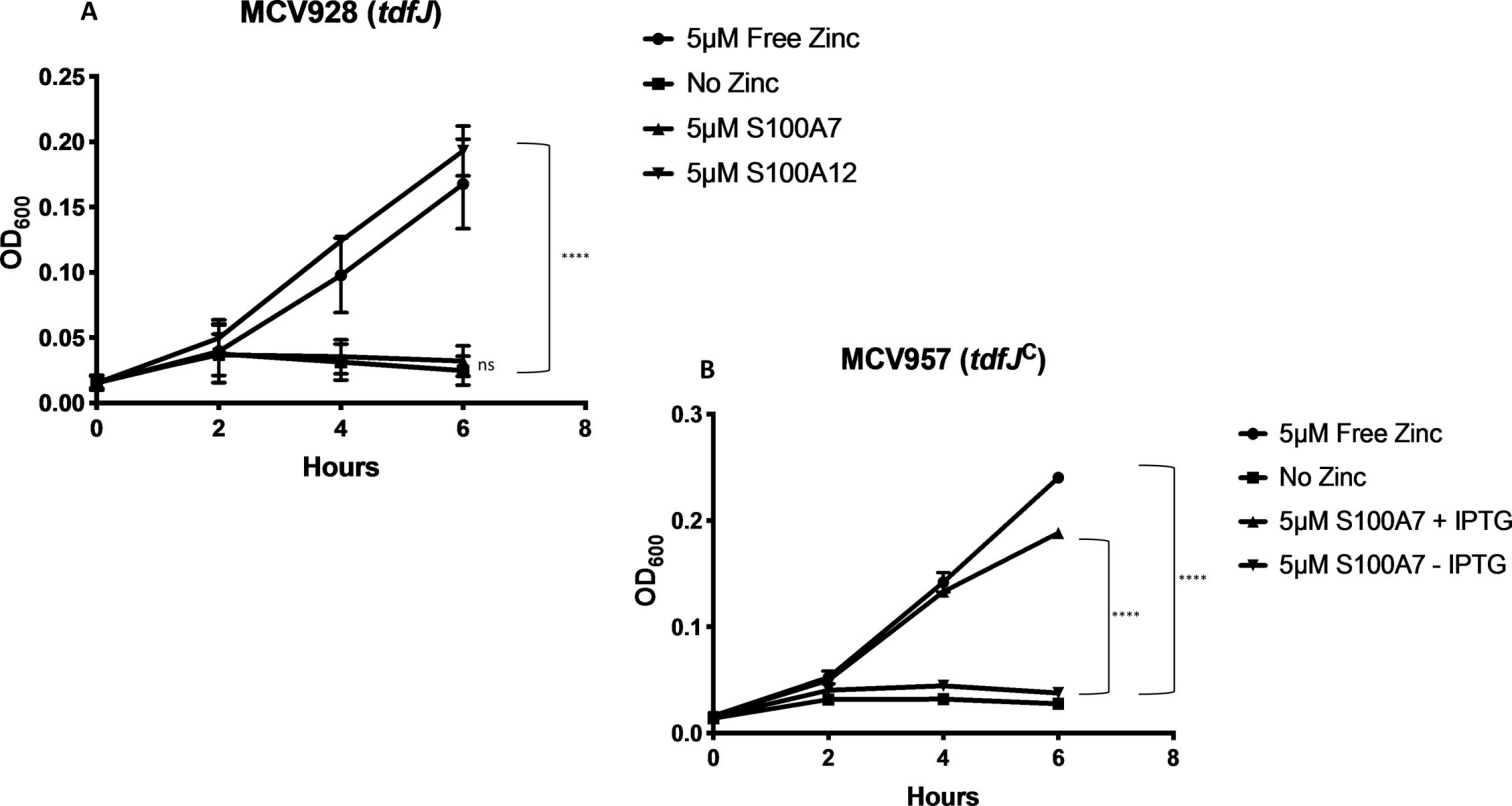
*Neisseria gonorrhoeae* requires a functional TdfJ to utilize S100A7 as a sole zinc source. (A) The *N*. *gonorrhoeae tdfJ* null mutant, MCV928, was grown in Zn-restricted CDM supplemented with growth premix containing ZnSO_4_, S100A7, S100A12, or no zinc. Growth was measured by OD_600_ readings every 2 h for 6 h total. (B) The *tdfJ* complemented strain, MCV957, was grown as described in (A), with S100A7 containing premixes further supplemented with either 1 mM IPTG or no IPTG. Both graphs demonstrate means and SD for N = 6 and N = 3 independent experiments for (A) and (B), respectively. A 2-way repeated measures ANOVA with Tukey’s correction was performed for all means, with significance at 6 h shown. *, P ≤. 05; **, P ≤ .005; ***, P ≤ .0005; ****, P ≤ .0001.

Since S100A7 growth support depended on ZnuA and TonB and adding S100A7 to cultures induced expression of TdfJ, we next sought to determine whether TdfJ contributed to use of S100A7 as a zinc source in the gonococcus. We grew a gonococcal mutant, strain MCV928 [61], which is incapable of producing TdfJ, in zinc-depleted conditions containing S100A7 and assessed growth over 6 hours. In the absence of TdfJ, S100A7 was completely unable to support the growth of *N*. *gonorrhoeae* as a zinc source (Fig 7A). Indeed, these samples were statistically indistinguishable from those containing no zinc. S100A12 fully supports growth of the *tdfJ* mutant strain, MCV928, indicating that its use as a zinc source is unrelated to TdfJ. To confirm that TdfJ was indeed responsible for the observed phenotype, we generated a *tdfJ*-complemented strain of MCV928, named MCV957, which was inducible by addition of isopropyl β-D-1 thiogalactopyranoside (IPTG). MCV957 was grown as described for MCV928 with and without IPTG. The induced sample of MCV957 completely recovered the growth phenotype in the presence of S100A7 as observed in the wild-type, while the uninduced sample remained indistinguishable from growth with no zinc (Fig 7B). These data in conjunction with that observed for *znuA* and *tonB* mutants suggest that the gonococcus uses the TdT TdfJ, followed by ZnuABC transporter in the inner membrane, to acquire zinc from S100A7 and overcome host-mediated zinc sequestration.

**Fig 8:**
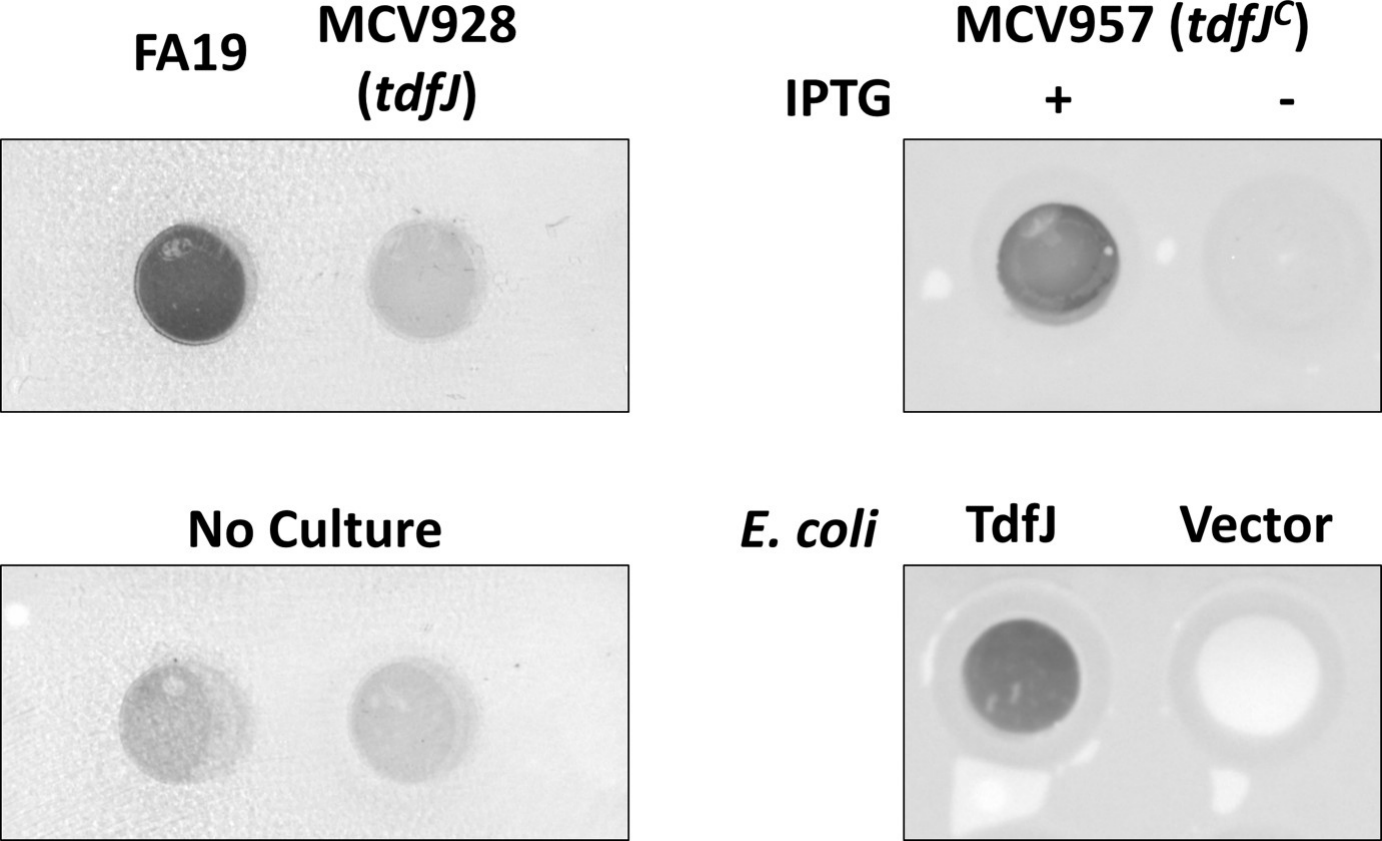
S100A7 binds to both gonococcal and recombinant TdfJ. *N*. *gonorrhoeae* strains FA19, MCV928 (*tdfJ*), and MCV957 (*tdfJ*^c^) with and without IPTG were grown as described in Zn-restricted CDM supplemented with 24 μM Fe(NO_3_)_3_ for 4 h before being applied to nitrocellulose in a dot blot apparatus at a standardized optical density (top row). *E*. *coli* strains containing pVCU313 for TdfJ production or carrying an empty pET-11a vector were grown in LB and induced for 4h before standardizing to the same optical density as used for gonococci and subsequently applied to nitrocellulose (bottom right). Samples were blocked and then probed for 1 h with HRP-labeled S100A7 diluted in blocker before colorimetric development. Wells lacking cells were probed with S100A7-HRP and developed to determine background signal (bottom left). Images are representative of N = 3 experiments.

To examine whether TdfJ bound directly to S100A7 for bacterial zinc acquisition, we grew strains FA19, MCV928, and MCV957 with and without IPTG in CDM treated with TPEN and Fe(NO_3_)_3_. Bacteria from cultures of a standardized density were then applied to a nitrocellulose membrane. This allowed whole-cells with an intact outer membrane to present surface proteins in their native configuration. These membranes were blocked and subsequently probed with horseradish peroxidase (HRP)-labeled S100A7. Only FA19 and the induced MCV957 showed detectable binding of S100A7–HRP (Fig 8). These results demonstrate that not only is wild-type *N*. *gonorrhoeae* capable of binding directly to S100A7, but that TdfJ is necessary for this interaction. Importantly, we recapitulated the binding of S100A7 in *E*. *coli* expressing recombinant TdfJ on its surface, showing that TdfJ expression is sufficient to mediate interaction with S100A7. We conclude that gonococcal TdfJ is necessary and sufficient to enable binding to S100A7.

### TdfJ internalizes zinc from S100A7

**Fig 9:**
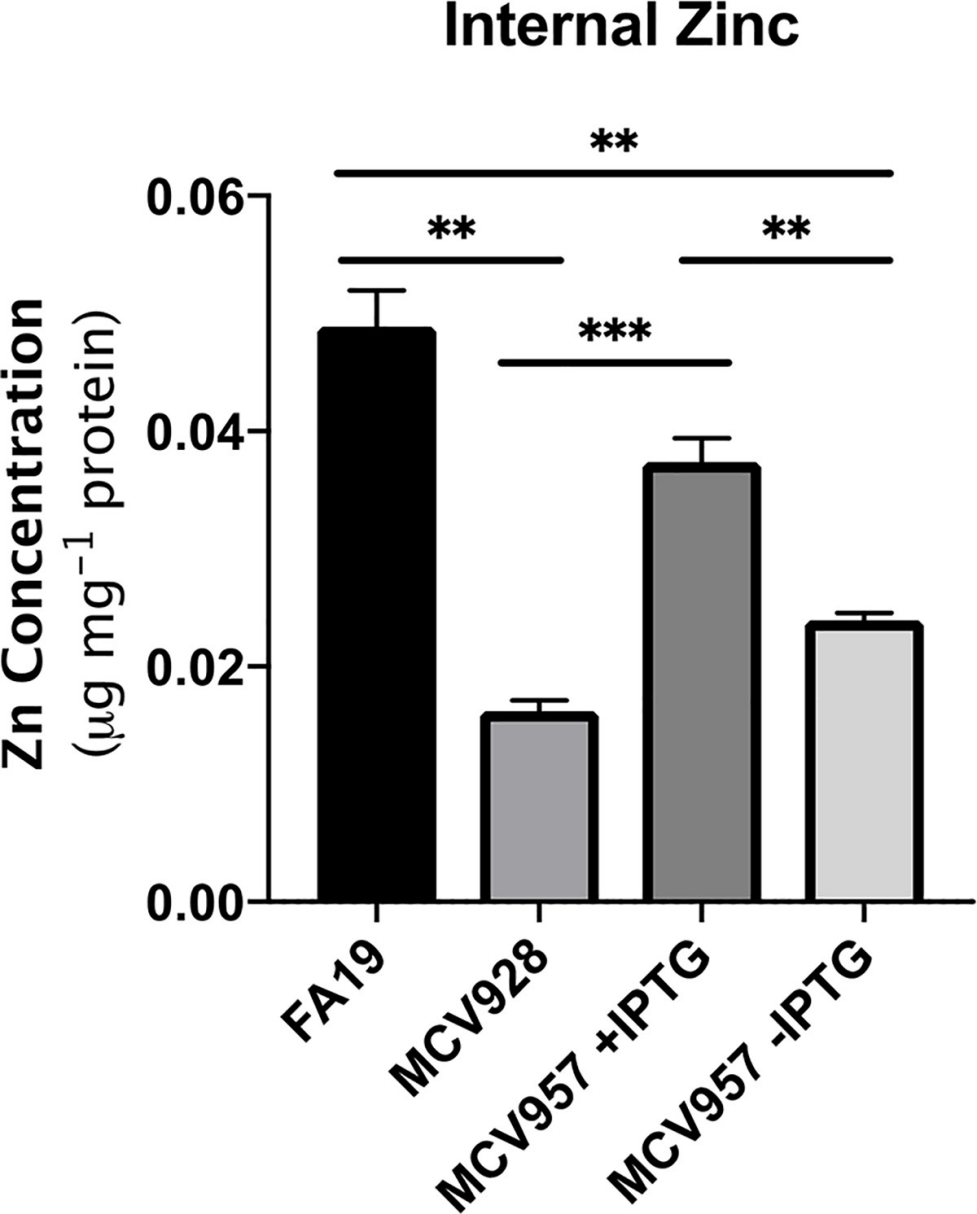
TdfJ internalizes zinc from S100A7. Strains FA19, MCV928 (*tdfJ*), and MCV957 (*tdfJ*^*C*^) with and without IPTG were grown in Zn-restricted rich medium supplemented with 2 μM partially-saturated S100A7. After 6 h cells were harvested and digested in concentrated TMG nitric acid prior to ICP-OES analysis to detect trace metals profile. Data shown are measured μg zinc/mg cellular protein, read at 213.857 nm, compared to a trace metals standard curve ranging from 5 to 4000 ppb. N = 3 independent cultures. *, P ≤. 05; **, P ≤ .005; ***, P ≤ .0005; ****, P ≤ .0001.

Having demonstrated that S100A7 both binds to TdfJ and supports growth as a zinc source, we next ascertained whether this pathway led to accumulation of zinc within *N*. *gonorrhoeae*. We grew strains FA19, MCV928, and MCV957 with and without IPTG in zinc-restricted medium in the presence of partially zinc-saturated S100A7 before quantifying the amount of zinc assimilation by using inductively coupled plasma optical emission spectrometry (ICP-OES). Emitted zinc ions were detected at 213.857 nm and then their concentrations were determined by comparison to a standard curve generated from serial dilutions of a 10 μg/mL trace metal standard (Inorganic Ventures). These concentrations were then standardized to reflect μg zinc per mg cellular protein (Fig 9). Strain FA19 internalized significantly more zinc from S100A7 than was detected for MCV928, indicating TdfJ is necessary for this process. IPTG-induced MCV957 similarly showed significantly more zinc assimilation than both MCV928 and uninduced MCV957, which agrees with our previous experiments that showed complementation of *tdfJ* recovers both gonococcal binding of and growth support by S100A7. These data demonstrate that gonococcal TdfJ interacts with S100A7 in a manner that facilitates zinc internalization by the pathogen.

### TdfJ interacts specifically with the human form of S100A7, and S100A12 does not compete for TdfJ binding

**Fig 10:**
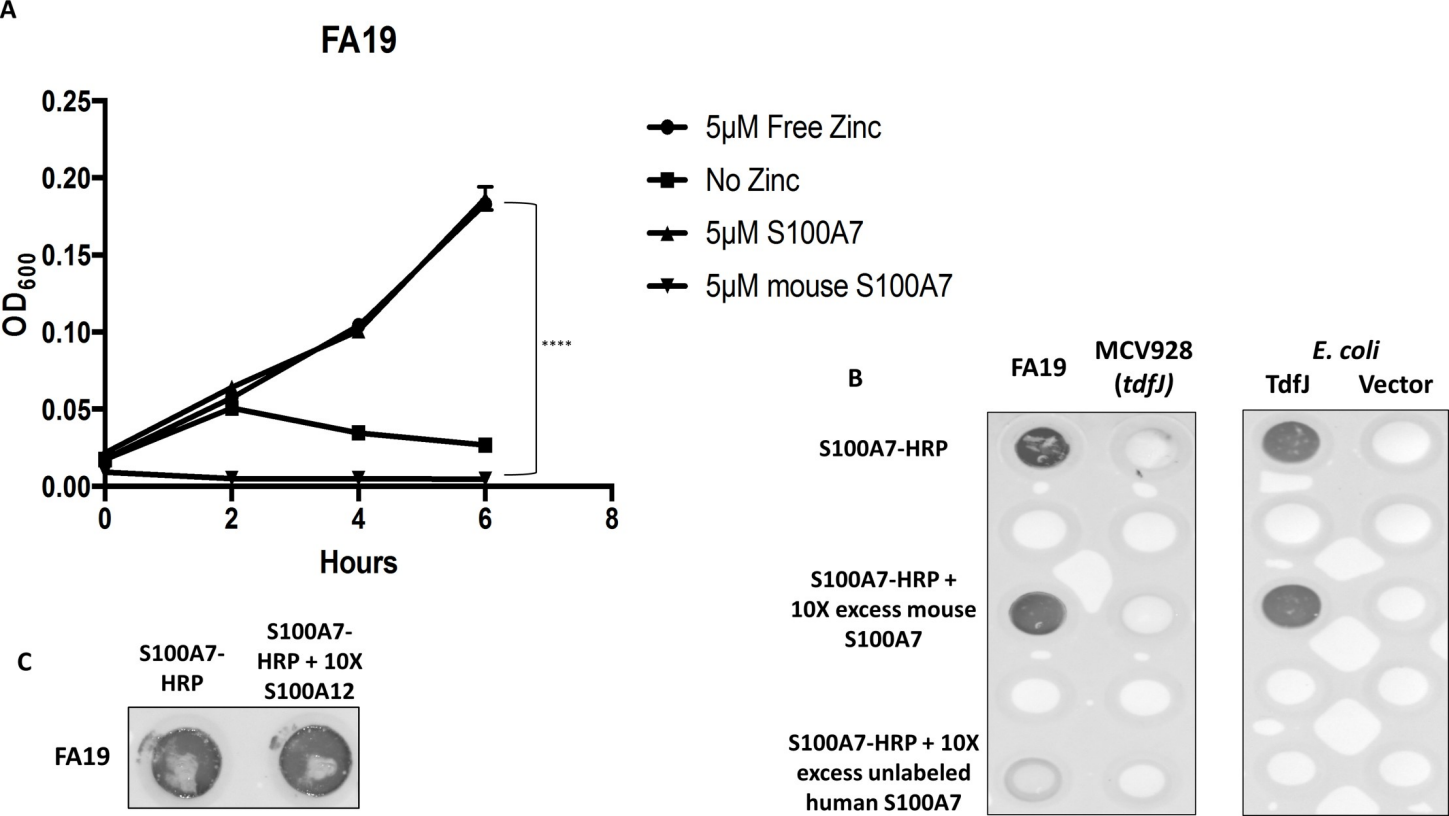
TdfJ utilization of and binding to S100A7 is specific for the human version of S100A7. (A) Strain FA19 was grown in Zn-restricted CDM supplemented with growth premix containing ZnSO_4_, human S100A7, mouse S100A7, or no zinc. OD_600_ readings were taken every 2 h for 6 h total. Mean and SD for N = 3 experiments are represented in the graph. A 2-way repeated measures ANOVA with Tukey’s correction was performed for all means, with significance at 6 h shown. *, P ≤. 05; **, P ≤ .005; ***, P ≤ .0005; ****, P ≤ .0001 (B) Strain FA19 and MCV928 (*tdfJ*) were grown in Zn-restricted CDM supplemented with 24 μM Fe(NO_3_)_3_ for 4 hours, after which equal culture densities were transferred to nitrocellulose in a dot blot apparatus. *E*. *coli* containing pVCU313 for TdfJ production or an empty pET-11a were grown in LB and induced for 4 h before being standardized to the same optical density used for gonococci and applied to nitrocellulose. Blots were then blocked and subsequently treated with HRP-labeled S100A7 either alone or with a 10-fold molar excess of unlabeled mouse or human S100A7 as competitor. Following this treatment blots were washed and colorimetric signal detection performed. (C) Strain FA19 was grown as described in part (B) before being standardized and dotted onto nitrocellulose. After blocking, blots were probed with either HRP-labeled S100A7 alone or in the presence of 10-fold molar excess of unlabeled S100A12, and signal was detected after washing. Blots shown are representative of N = 3.

*N*. *gonorrhoeae* is an exclusive human pathogen, and as such many interactions are specific for the human host [62]. We set out to determine whether gonococcal binding to and utilization of S100A7 shared this characteristic. The mouse version of S100A7 (mS100A7, Abbexa) was loaded with zinc at the same saturation as used for the experiments conducted with human S100A7 and presented to wild-type FA19 cells as a sole zinc source in otherwise zinc-depleted media. mS100A7 was completely unable to support growth of gonococci, demonstrating optical densities even lower than those samples given no zinc source (Fig 10A). As mS100A7, like the human version, sequesters zinc, it is likely that this further diminished growth phenotype is due to additional, effective zinc chelation by mS100A7 during the assay as there is no mechanism present for the gonococcus to circumvent this.

In addition to growth restriction, we tested the ability of gonococci and recombinant *E*. *coli* to physically bind mS100A7 by performing competition assays. FA19, MCV928, and MCV957 with and without IPTG, or *E*. *coli* expressing *tdfJ* or an empty vector were applied to nitrocellulose in standardized amounts before blocking and probing with S100A7-HRP. Simultaneously with the ligand, we added 10-fold molar excess of either mS100A7 or unlabeled human S100A7 as competitor. As before, only those cultures presenting TdfJ on the surface generated any signal from S100A7-HRP, and only samples treated with human competitor demonstrated signal reduction; mS100A7 did not compete for binding of gonococcal TdfJ (Fig 10B). Likewise, when unlabeled S100A12 was included at 10-fold molar excess to S100A7-HRP, it was similarly unable to compete for binding and this resulted in no signal reduction (Fig 10C). These data are consistent with the conclusion that the TdfJ-S100A7 interaction, like other gonococcal surface structure-host ligand interactions, is specific to the human protein, emphasizing the nature of *N*. *gonorrhoeae* as an obligate human pathogen. Moreover, they support the conclusion that TdfJ recognizes S100A7 specifically, instead of simply having affinity for the human S100s in general.

### Mutant S100A7 unable to bind zinc is deficient for TdfJ-mediated growth support

**Fig 11:**
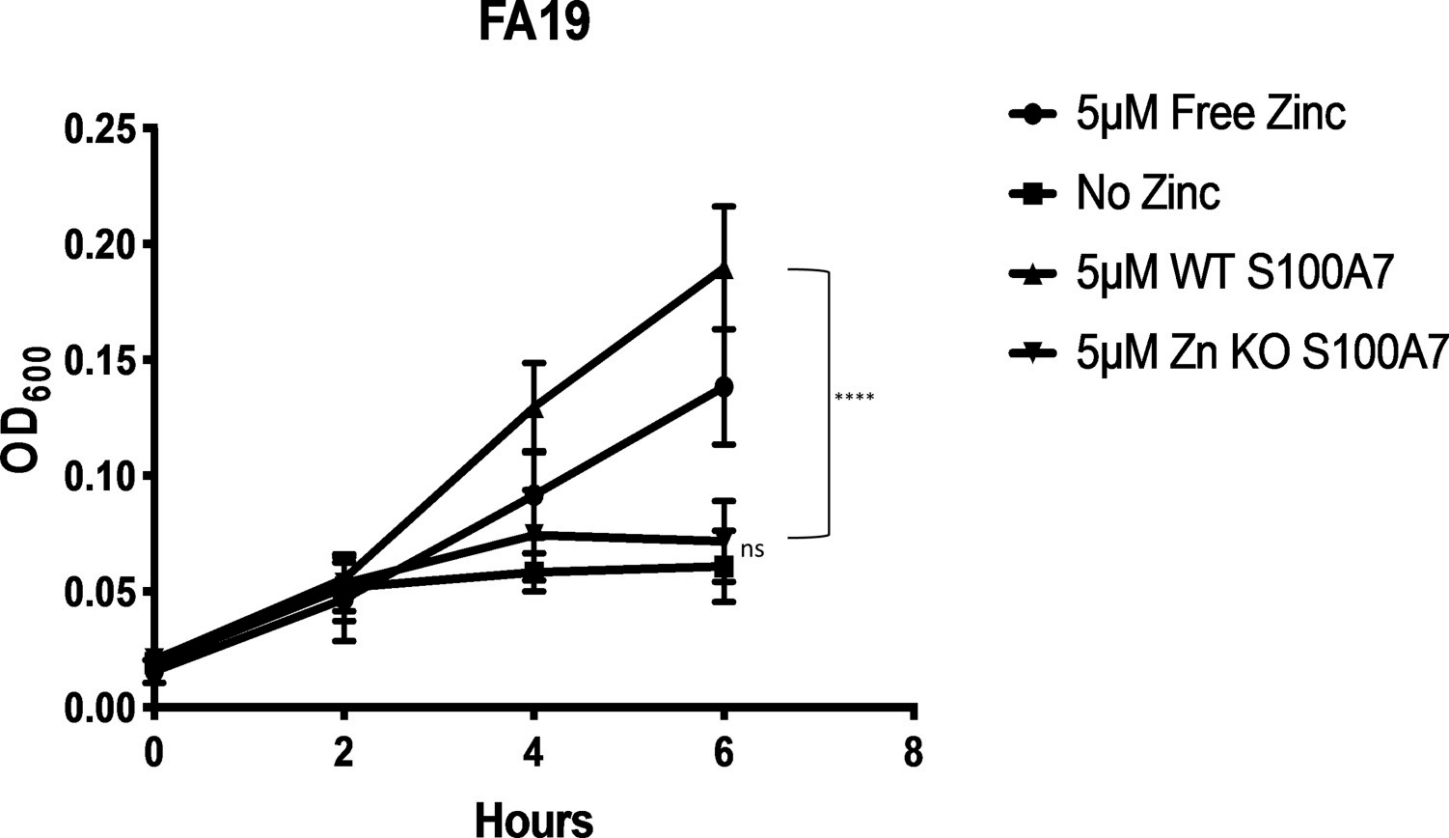
Mutated S100A7 unable to bind zinc is deficient in TdfJ-dependent growth support. Both wildtype S100A7 and the S100A7 mutant incapable of binding zinc were mixed with ZnSO_4_ at a 2:1 molar ratio and incubated with end-over-end mixing for 18 h to allow any zinc loading to occur. After this, both mixtures were dialyzed against Chelex-100-treated dialysis buffer to remove any unbound ions before being utilized in growth premix. Strain FA19 was grown in Zn-restricted CDM supplemented with premix containing either wildtype or mutant S100A7, with OD_600_ readings taken every 2 h for 6 h total. The graph demonstrates means and SD for N = 3 independent experiments. A 2-way repeated measures ANOVA with Tukey’s correction was performed for all means, with significance at 6h shown. *, P ≤. 05; **, P ≤ .005; ***, P ≤ .0005; ****, P ≤ .0001.

Lastly, we were interested to see if zinc sequestered by S100A7, as opposed to unchelated zinc or other transition metals which are freely available in the media, is specifically what allows growth support of the gonococcus in zinc-limited conditions. To test this, we grew strain FA19 in zinc-depleted CDM supplemented with an H87N+H91N double mutant S100A7 (Zn KO S100A7), which is unable to bind zinc. The zinc affinity of Zn KO S100A7 was investigated by isothermal titration calorimetry, which verified that it is devoid of zinc binding activity, whereas the zinc affinity of the wild-type S100A7 is in the nM range (S1 Fig). Furthermore, bacterial growth inhibition assays [35] showed that this mutant is much less effective than the wild type protein at suppressing bacterial growth, as a by-product of its inability to sequester metals. Prior to growth assays, Zn KO S100A7 was mixed with ZnSO_4_ for sufficient time to allow zinc loading as for the wild-type protein, before being dialyzed against buffer containing Chelex-100 to remove unbound metals. As a control, wild-type S100A7 was prepared in an identical manner and included in the assay for comparison. When presented to gonococci in zinc-deplete conditions, Zn KO S100A7 did not support bacterial growth, and was statistically indistinguishable from samples treated with no zinc, while samples grown with wild-type S100A7 retained their previously-observed growth phenotypes (Fig 11). From these data we conclude that the ability of S100A7 to chelate zinc is essential for its ability to support growth of the gonococcus in a TdfJ-dependent manner.

## Discussion

Nutritional immunity, the sequestration of essential nutrients by hosts to hamper invading pathogens, and the strategies deployed by bacteria to overcome this defense, has become a prevalent theme in the evolving host-pathogen struggle. Iron, zinc, manganese, and other transition metals are key nutrients for bacterial survival and thus infection [25, 26]. *N*. *gonorrhoeae* is particularly capable of overcoming nutritional immunity using non-traditional mechanisms. Unlike many other pathogens, *N*. *gonorrhoeae* does not produce any siderophores and therefore does not directly scavenge free iron from its environment. Instead, it utilizes an arsenal of TonB-dependent transporters that bind directly to host nutritional immunity factors including transferrin, lactoferrin, and hemoglobin and strip them of their iron cargo, effectively co-opting them for support of infection [13]. More recently, it has become clear that this phenomenon is not restricted to only iron, as both the gonococcus and meningococcus can use other TdTs to acquire zinc by a similar strategy [17, 18, 51]. Considering the high level of structural and functional conservation of the gonococcal TdTs, we hypothesized that the uncharacterized TdTs likewise play an important role in the ability of *N*. *gonorrhoeae* to survive and grow in the context of human nutritional immunity factors. Critically, recent studies have shown these transporters to be promising targets for vaccine development [63, 64].

The S100 family of EF-hand calcium binding proteins play several essential roles in vertebrates, including sequestration of transition metals to starve invading bacteria of key nutrients [42]. Calprotectin, S100A12, and S100A7 have been shown to exert suppressive activity against numerous pathogens, including *E*. *coli*, *S*. *aureus*, *P*. *aeruginosa*, *Shigella flexneri*, *Helicobacter pylori*, and *C*. *albicans* [40–47]. This report and our previous work [18] demonstrate that *N*. *gonorrhoeae* is able to overcome nutritional immunity by utilizing these otherwise antimicrobial proteins as zinc sources. Other pathogens including *Aspergillus fumigatus*, *Salmonella enterica* serovar Typhimurium, and *Acinetobacter baumannii* have shown the ability to circumvent S100-mediated zinc restriction by employing their own zinc acquisition systems [34, 65]. The high-affinity zinc uptake system ZnuABC, an inner-membrane ABC transporter, has been a consistent factor present in Gram-negative bacteria that employ these acquisition systems.

*N*. *gonorrhoeae* is no exception to this phenomenon, as S100A12 and S100A7 utilization explicitly depends on the function of this ABC transporter. Until recently, however, the mechanism of zinc passage through the outer membrane in *N*. *gonorrhoeae* was unknown. Jean et al [18] showed that *N*. *gonorrhoeae* utilizes TdfH for binding Calprotectin and consequent acquisition of zinc. Moreover, the meningococcal TdfJ homologue, ZnuD, has demonstrated binding of extracellular zinc [52], and both ZnuD and TdfJ are regulated by zinc via the regulator Zur [66], which is consistent with the expected phenotype of zinc importers. Gonococcal TdfH and meningococcal ZnuD have been shown to contribute to neisserial growth in zinc-deplete conditions [17, 18, 51]. For these reasons, we sought to evaluate S100A12 and S100A7 as potential ligands for other TdTs, specifically TdfJ.

Our data show that growth support by S100A7 not only requires ZnuABC, but growth is also abrogated in the absence of TonB, suggesting the participation of one of the TdTs in its utilization. Indeed, S100A7 can only serve as a sole zinc source when *N*. *gonorrhoeae* produces a functional TdfJ. *In vitro*, this interaction, like the other TdT-mediated interactions, allows the gonococcus to overcome metal sequestration and grow effectively. Furthermore, we found in the current study that gonococci grown in the presence of S100A7 are enriched for internal zinc, which depends on TdfJ. Finally, we sought to clarify whether zinc was truly the nutritional cargo of S100A7 responsible for this interaction, and not some other adventitious metal present in the protein or media. A His-Asn double mutant S100A7, with substantially reduced zinc affinity, was defective in growth support under zinc-depleted conditions, suggesting that zinc presence on S100A7 is indeed required for growth support.

After establishing that S100A7 supports gonococcal growth in a TdfJ-dependent manner, we sought to establish whether these two proteins directly bound to each other, as is the case for the other characterized gonococcal TdTs and their ligands. When grown in conditions ideal for TdfJ production, wild-type *N*. *gonorrhoeae* and induced *tdfJ*-complemented strains were able to directly bind S100A7, while mutant and uninduced strains not capable of producing TdfJ demonstrated no binding, suggesting a direct protein-protein interaction. Moreover, the growth conditions used are also ideal for production of the related zinc transporter TdfH, so this protein was present on the outer membrane as well. The lack of S100A7 binding to *tdfJ* mutant strains that still express a functional TdfH suggests that this binding interaction is indeed specific for TdfJ. We considered the possibility that inactivating the *tdfJ* gene, which encodes a membrane-embedded protein, may have generated unexpected, unintended alterations to the outer membrane as a whole and thus potentially confound binding experiments. To address this, we probed recombinant *E*. *coli* expressing either gonococcal TdfJ or containing an empty vector and found that TdfJ presence alone is sufficient for S100A7 binding, thus confirming their interaction, and aligning with previously described binding experiments between TdfH and Calprotectin [18].

As an obligate human pathogen, *N*. *gonorrhoeae* utilizes many virulence factors that are restricted to the human host [62]. We demonstrated in the current study that both gonococcal and recombinant TdfJ bind only to the human version of S100A7, and do not interact with the mouse version. While mouse S100A7 shares only 34% sequence identity with its human counterpart, its expression patterns, structure, and function are similar in both mice and humans [67, 68]. When present at 10-fold molar excess, mS100A7 shows no ability to compete for binding when human S100A7 is present. We showed also that when presented to the gonococcus as a zinc source, mS100A7 is unable to support gonococcal growth, and indeed seems to directly hinder growth. As noted in the methods section, mS100A7 showed detectable amounts of zinc already present upon reconstitution of the commercial protein. This means that our own addition of zinc probably exceeded our target of 25% saturation, but we do not view this as a detriment to the growth assays. Because the hypothetically more zinc-rich protein still failed to support gonococcal growth, it strengthens our conclusion that the gonococcus cannot use mouse S100A7 as a zinc source.

Our finding that TdfJ is both induced by iron and repressed by zinc agrees with previous studies, and here we showed that these two regulators have additive effects on gene expression. When considered independently, downregulation by zinc had a slightly more pronounced effect than induction by iron, which is consistent with the expected phenotype of a zinc transporter. It is not expected that TPEN presence played any role in unintended chelation of iron from samples where both were present. Among the gonococcal Tdfs, only TdfJ is induced by iron; TdfH is not regulated by iron [18], and two other proteins TdfF and TdfG are in fact iron repressed [59, 69]. Despite the apparent regulation of TdfJ by iron, our experiments revealed no clear functional relationship between the two, so the reason for this coordinate regulation is left to speculation. One possible explanation is that TdfJ is the preferred mediator of zinc uptake for *N*. *gonorrhoeae* when iron is replete; the *tdfJ* gene is found across all of genus *Neisseria* and its protein products are highly conserved. Unlike TdfH, TdfJ is able to bind directly to free zinc, which is then internalized [52]. Furthermore, these observations are substantiated by key regulatory motifs in the *N*. *gonorrhoeae* genome. Pawlik et al [66] identified the sequence of a putative Zur binding motif (Zur box) for meningococci, and we found, via pattern location software (http://www.cmbl.uga.edu/software/patloc.html), that a 100% match for this motif (TGTTATATAATAACA) is located within the promoter region of *tdfJ*. We used the same software to search for putative Fur boxes upstream of *tdfJ* and none were identified within 1.5 kilobases from the start of the gene. This agrees with the observation that iron presence enhanced TdfJ production rather than repressing it, as would be expected for the typical function of Fur. Paradoxically, previous reports have shown that the Fur protein binds within the promoter region of *tdfJ*, but the precise binding site was not mapped [70]. Therefore, at this time the mechanism of iron regulation of *tdfJ* is unresolved, but has been observed to occur at the transcriptional level [71]. TdfH and TdfF production is limited to pathogenic *Neisseria* but not commensal species [72], and TdfG is restricted almost exclusively to *N*. *gonorrhoeae* [69]. Moreover, Tdfs G and F have as yet not demonstrated any zinc-related phenotype.

In this report, we define a novel interaction between a gonococcal TdT (TdfJ) and the human zinc-binding protein S100A7, wherein TdfJ on the gonococcal surface directly binds S100A7 and utilizes if for zinc internalization, thus overcoming one type of host-mediated nutrient restriction. This interaction, limited to the human host, may provide a clear selective advantage to *N*. *gonorrhoeae* in the context of infection, especially at the mucosal epithelium, a biologically relevant niche for the gonococcus where S100A7 is enriched [43, 44]. The S100A15 protein is highly similar to S100A7 and has also demonstrated metal-sequestering antimicrobial effects [73], so it is possible that in these or other host tissues, it may function alongside or in concert with S100A7, but its relationship to *N*. *gonorrhoeae* and TdfJ was not explored in the current study. We also show that another zinc-binding protein, S100A12, supports gonococcal growth as a sole zinc source by an as-yet-uncharacterized manner dependent upon ZnuABC. While the mechanism for S100A12 utilization is not yet clear, TonB-independent siderophore-iron uptake has been observed [61, 74]. It is conceivable that ZnuABC contributes to gonococcal zinc uptake and infection in a manner independent of the TdTs, as is seen in, for example, pathogenic *E*. *coli*, *Salmonella* and *Vibrio cholerae* [75–77]. While it is not immediately apparent how the periplasmic ZnuA protein can extract zinc from extracellular S100A12, previous reports have suggested that ZnuABC systems can help bacteria overcome zinc restriction by the S100 protein Calprotectin in the absence of a TonB-dependent transporter to shepherd the metal through the outer membrane [78]. In light of these findings, we suggest that TdfJ is a promising vaccine target for this important pathogen, and we also note the fact that ZnuD has received consideration for the same purpose in meningococcus [54]. TdfJ is exposed on the bacterial surface and is not subject to the high-frequency phase and antigenic variation that has disqualified so many other surface structures from consideration. TdfJ may contribute to *in vivo* survival of the gonococcus by utilization of S100A7, which is highly upregulated in the epithelia. We also note that ZnuD has been shown to participate in interactions with epithelial cells [79]. Finally, TdfJ is ubiquitously produced across the *Neisseria* species, including the commensals from which gonococci and meningococci frequently acquire resistance factors, and which themselves may be opportunistic pathogens [80].

To summarize, we report a novel interaction between *N*. *gonorrhoeae* and its human host, which allows the gonococcus to overcome the innate immune mechanism of nutrient starvation. Considering the potential importance of this strategy for infection, alongside TdfJ’s presumed potential as a promising vaccine target, further characterization of the TdfJ-S100A7 interaction is underway in order to clarify the importance of TdTs as virulence factors for *N*. *gonorrhoeae*.

## Materials and methods

### Bacterial growth conditions

*E*. *coli* strains were cultured in Luria-Bertani (LB) medium supplemented with antibiotics at the following concentrations: 100 μg/mL for carbenicillin, 34 μg/mL for chloramphenicol, and 50 μg/mL for spectinomycin. Strains of *N*. *gonorrhoeae* were maintained on GC medium base (Difco) agar with Kellogg’s supplement I [81] and 12 μM Fe(NO_3_)_3_ (GCB plates) at 37°C at 5% CO_2_. For the *znuA* mutant strain, MCV951, this media and all others were supplemented with 5 mM D-mannitol (Sigma) [57, 58]. For growth of *N*. *gonorrhoeae* in liquid culture, both rich and defined media were used, and zinc restriction was accomplished by addition of TPEN (Sigma) at the minimum amount needed to inhibit growth of the wild-type strain FA19. Zinc-restricted growth in rich media was accomplished by inoculating GC broth treated with supplement I, 12 μM Fe(NO_3_)_3_, and 10 μM TPEN with colonies grown on GCB plates. These cultures were grown at 37°C + 5% CO_2_ with shaking until log phase, at which point they were back diluted to the same optical density with the same medium and treated with 2 μM S100A7 loaded with 1 μM ZnSO_4_ and 1 mM IPTG, if needed. For growth in defined medium, colonies from GCB plates were used to inoculate chemically defined medium (CDM) that had been treated with Chelex-100 resin (Bio-Rad). These cultures were grown as described until log phase before back dilution and treatment with 24 μM Fe(NO_3_)_3_, 5 μM S100A7, 10 μM ZnSO_4_, and/or 1 or 5 μM TPEN for preparation of whole cell lysates (WCLs), or treated with growth premix (described below) for zinc-dependent growth assays. For WCLs, growth was allowed to proceed for 6 hours as described before lysates of a standardized density were collected and subjected to SDS-PAGE and western analysis.

### Production of S100A12, S100A7, Mouse S100A7 and Zn KO S100A7

S100A12 was produced from a pGEMEX expression vector provided by Professor Claus Heizmann and purified as described elsewhere [49]. S100A7 was produced from a pET-22b expression vector provided by Professor Joachim Grötzinger (pET-22b-pso) using the protocol described by Grötzinger and coworkers [82]. The Zn KO S100A7 (H87N, H91N double mutant) was generated by Q5 mutagenesis (New England Biolabs). In brief, the pET-22b S100A7 H87N+H91N plasmid was used to transform BL21 (DE3) *E*. *coli* following standard procedures. When the OD_600_ reached 0.6, cells were induced at 37°C by the addition of 1 mM IPTG and allowed to grow 4 hours post-induction. Cells were harvested by centrifugation and were resuspended in lysis buffer (50 mM Tris pH 8.0, 100 mM NaCl, 1 mM EDTA, 1 mM PMSF, 0.5% Triton X-100). Cells were then sonicated and centrifuged for 20 minutes. The supernatant was filtered and loaded onto a SepharoseQ column (GE). After loading, the column was washed with 3 CV buffer A (20mM Tris pH 8.0) and eluted with a gradient (10 CV, 0 -> 1 M) to buffer B (20 mM Tris pH 8.0, 1 M NaCl). Relevant fractions were pooled, concentrated, and loaded onto an S75 column. Protein was eluted with 1 CV S75 buffer (20 mM Tris pH 8.0, 100 mM NaCl). Relevant fractions were pooled, flash frozen, and stored at -80°C. To confirm that no contaminating zinc was present in these preparations, ICP-MS experiments were performed on WT and mutant S100A7 produced for this study, which showed that WT proteins had less than 0.1 molar equivalents of Zn, while Zn KO S100A7 had less than 0.01 molar equivalents. Mouse S100A7 was purchased as lyophilized protein from Abbexa and was free from buffer or chelating agents upon receipt. Prior to use in experiments, the protein was reconstituted in buffer consisting of 20 mM Tris pH 8.0, 100 mM NaCl, 20 mM β-Mercaptoethanol, and 1 mM CaCl_2_. ICP-MS experiments were also performed on this protein, which revealed 0.4 molar equivalents of zinc already present prior to our own zinc loading. Comparable amounts of nickel were also detected in these preps, likely due to the use of Ni-NTA during protein purification. The affinity of wild-type and Zn KO S100A7 for zinc was determined by isothermal titration calorimetry (S1 Fig) as described elsewhere [83].

### Metal loading of proteins and preparation of growth premix

Human transferrin (Sigma) was dissolved in a mixture of 40 mM Tris, 150 mM NaCl, and 10 mM NaHCO_3_ at pH 8.4 (initial buffer) before being incubated with FeCl_2_ to result in ~30% Fe saturation. After incubation, this solution was dialyzed against excess 40 mM Tris, 150 mM NaCl, and 20 mM NaHCO_3_ at pH 7.4 (dialysis buffer) to remove unbound iron. Bovine apo-transferrin (Sigma) was dissolved in initial buffer and not treated with any metal before dialysis, such that it could sequester any residual iron in the final mix. S100 proteins were maintained in their own unique buffers after being purified as described. For zinc loading, the dimeric S100 proteins were incubated with ZnSO_4_ at a 2:1 molar ratio (dimer:zinc) to accomplish ~25% zinc loading. These ingredients plus TPEN and phosphate buffered saline, pH 7.4 (PBS) were combined into a concentrated growth “premix” which was used to restrict *N*. *gonorrhoeae* to defined sources of iron and zinc for growth in microtiter dishes. When diluted by liquid culture, final concentrations in the premix were as follows: 7.5 μM 30%-Fe human transferrin, 3 μM bovine apo-transferrin, 5 μM 25%-Zn S100 protein, and 1 μM TPEN. For a positive zinc control, TPEN was omitted and S100 proteins were replaced with 5 μM ZnSO_4_, and for a negative control all zinc sources were omitted. To accommodate the *tonB* mutant strain, MCV650, which is unable to utilize human transferrin as an iron source [60], both human and bovine transferrin were omitted from the recipe and replaced by a final concentration of 3 μM Fe(NO_3_)_3_.

### Gonococcal mutant construction

All primers used in this study were produced by New England Biolabs and can be found in Table 1. All strains and plasmids utilized in this study are summarized in Table 2. All restriction endonucleases were acquired from New England Biolabs. For construction of the *znuA* mutant strain (MCV951), primers oVCU865 and oVCU866 were used to amplify a region of the *znuA* gene from chromosomal DNA isolated from strain FA19. The resulting PCR product was purified and ligated into the EcoRI site of pVCU403 [84] via the In-Fusion cloning method (Clontech). This ligation mixture was used to transform TOP10 (Invitrogen) *E*. *coli*, and transformants were selected on 100 μg/mL carbenicillin and confirmed by PCR to generate pVCU550. To disrupt the *znuA* gene, an Ω cassette (Spc^R^ Str^R^) was ligated into the BmgB1 site of the *znuA* gene from pVCU550 using T4 DNA ligase. This ligation was used to transform TOP10 *E*. *coli* and transformants were selected on 50 μg/mL spectinomycin and confirmed by PCR. The resulting plasmid, pVCU551, was linearized with SspI and used to transform a piliated polulation of FA19, creating strain MCV951, which was confirmed by PCR. Recovery of these transformants required GCB plates supplemented with 25 μM each of Zn and Mn plus 5 mM D-mannitol, in addition to selection on 50 μg/mL spectinomycin. To create an IPTG-inducible *tdfJ* complement, primers oVCU967 and oVCU968 were used to amplify the full *tdfJ* gene from FA19, which was cloned via In-Fusion into pVCU234 [85] that had been linearized with XmaI and XhoI and used to transform TOP10 *E*. *coli*. pVCU234, a modified version of pKH37 [86] inserts into an ectopic location in the gonococcal chromosome between the *aspC* and *lctP* loci. Transformants were selected on 34 μg/mL chloramphenicol and confirmed by PCR and sequencing. This plasmid, pVCU554, was linearized with PciI and used to transform piliated MCV928, and transformants were selected on 1 μg/mL chloramphenicol. The resulting strain, MCV957, was confirmed by PCR.

**Table 1:** Primers used in this study.

**Primer**	**Sequence**	**Purpose**
oVCU865	CTCTAGAGGATCCCCCACCTCAAACTTACCCTTAT	*znuA* Disruption Fragment Forward
oVCU866	CCATGATTACGAATTGCCGAGTGCGTCGGAATA	*znuA* Disruption Fragment Reverse
oVCU967	TTAAAAGGAGCCCGGGATGCGACGAGAAGCCAAAATGG	*tdfJ* Complement Forward
oVCU968	CGGGCCCCCCCTCGAGAAACTTCACGTTTACGCCGCC	*tdfJ* Complement Reverse
oVCU173	GGCACCCAGCCTGCGCGAGCAGGGG	Ω Cassette Outward (Left)
oVCU174	CGCAACATCCGCATTAAAATCTAGCGAGGG	Ω Cassette Outward (Right)
oVCU900SM	CACGAACCCAGTGGACATAA	Ω Cassette Forward
oVCU901SM	GGGACAACGTAAGCACTACA	Ω Cassette Reverse
oVCU816	CTAGGCACCCCAGGCTTTACAC	pKH37 Sequencing
oVCU896	CAGACCGTTCAGCTGGATATT	*cat* Marker Forward
oVCU897	CCTTGTCGCCTTGCGTATAA	*cat* Marker Reverse
ZnKO A7 F	CCGATTATAATAAACAGAGCAATGGTGCCG	Q5 Mutagenesis S100A7
ZnKO A7 R	CGGCACCATTGCTCTGTTTATTATAATCGG	Q5 Mutagenesis S100A7

**Table 2:** Strains and plasmids used in this study.

**Strain/Plasmid**	**Genotype/Purpose**	**Reference**
*E*. *coli* Strains		
TOP10	F- *mcrA* Δ(*mrr-hsdRMS-mcrBC*) φ80*lacZ*ΔM15 Δ*lacX74 nupG recA1 araD139* Δ(*ara-leu*)7697 *galE15 galK16 rpsL*(Str^R^) endA1 λ^-^	Invitrogen
BL21 (DE3)	F^−^*ompT gal dcm lon hsdS*_*B*_(*r*_*B*_^–^*m*_*B*_^–^) λ(DE3 [*lacI lacUV5*-*T7p07 ind1 sam7 nin5*]) [*malB*^+^]_K-12_(λ^S^)	NEB
OverExpress C41 (DE3)	F^−^*ompT gal dcm hsdS*_*B*_(r_B_^-^ m_B_^-^)(DE3)	Lucigen
*Gonococcal* Strains		
FA19	Wildtype	55
MCV928	FA19 *tdfJ*::Ω	61
MCV650	FA19 *tonB*::Ω	59
MCV951	FA19 *znuA*::Ω from pVCU551	This study
MCV957	MCV928 transformed with pVCU554	This study
Plasmids		
pET-11a	Expression vector	Novagen
pET-22b	Expression vector	Novagen
pET-22b-pso	pET-22b containing S100A7 coding sequence	82
pUC18	Cloning vector	Invitrogen
pVCU403	pUC18 + GC uptake sequence	84
pKH37	Complementation vector	86
pVCU234	pKH37 + Shine-Dalgarno Sequence	85
pVCU313	pBAD TOPO + *tdfJ*	87
pGEMEX	Expression vector containing S100A12 coding sequence	Heizmann
pVCU550	pVCU403 + *znuA* fragment	This study
pVCU551	pVCU550 + Ω cassette (*znuA*::Ω*)*	This study
pVCU554	pVCU234 + *tdfJ*	This study

### Western blotting

Whole-cell lysates of gonococci were harvested by pelleting cultures of a standardized optical density (100 Klett units in 1 mL culture) and resuspending cells in 2X Laemmli solubilizing buffer before storage at -20°C. Immediately preceding use, samples were thawed, mixed with 5% β-mercaptoethanol, and boiled for 5 minutes. Protein samples were separated on a 7.5% polyacrylamide gel before transfer to nitrocellulose. Blots were stained with Ponceau S to verify equal protein sample loading. To detect TdfJ, blots were blocked in 5% (w/v) nonfat dry milk dissolved in low-salt Tris-buffered saline (TBS, 150 mM NaCl) + .05% Tween 20. To generate polyclonal antibodies recognizing TdfJ, peptides from predicted extracellular loops 2 and 6 were synthesized by New England Peptide, Inc., conjugated to keyhole limpet hemocyanin, and used to immunize guinea pigs. [87] For western blots, this serum was diluted in blocker and used to probe blots for 2 hours at room temperature. Following washes with low-salt TBS, secondary antibodies conjugated to horseradish peroxidase (Invitrogen) were diluted in blocker and applied to blots for 1 hour at room temperature. Blots were then washed and developed using SuperSignal West Dura Extended Duration Substrate (Thermo) and imaged on a Bio-Rad ChemiDoc Gel Imaging System. As these are novel α-TdfJ antibodies, a full-sized western blot demonstrating the specificity of the antibodies is shown in S2 Fig.

### Growth with zinc-loaded S100 proteins

Gonococcal strains were grown in zinc-restricted CDM, as described, until log phase, at which point cultures were diluted to a standard optical density of OD_600_ = .02 and added to the wells of microplates pre-treated with growth premixes prepared as described and 1 mM IPTG, if needed. OD_600_ measurements were recorded using a Molecular Devices Vmax Microplate Reader at 0, 2, 4, and 6-hour time points. For strain MCV951, samples at the 6-hour mark were collected and serially diluted in sterile PBS before being spotted onto GCB plates supplemented with 5 mM D-mannitol. These plates were incubated for 24 hours at 37°C at 5% CO_2_ before colonies were enumerated and CFU/mL calculated.

### Whole-cell S100A7 binding and competition assays

S100A7 was conjugated to HRP using the ab102890 (Abcam) conjugation kit according to the manufacturer’s instructions. Protein concentration was then verified by bicinchoninic acid (BCA) assay. Gonococcal strains were grown in zinc-restricted CDM as described for 4 hours before cultures standardized to an optical density equivalent to 10 Klett units in 1 mL were applied to nitrocellulose using a dot blot apparatus (Bio-Rad). Membranes were blocked in 5% (w/v) nonfat dry milk dissolved in low-salt TBS before incubation for 1 hour with 0.2 μM S100A7-HRP diluted in blocker. Membranes were washed with low-salt TBS and signal developed with a Metal Enhanced DAB Substrate Kit (Thermo). For recombinant *E*. *coli*, OverExpress C41 (Lucigen) strains expressing *tdfJ* (pVCU313) or containing an empty pET-11a vector were grown as described until OD_600_ = ~.5 before induction with .02% arabinose or 1 mM IPTG, respectively, which was allowed to proceed for 4 hours. Cultures were then standardized to the same densities used for gonococci and added to nitrocellulose for S100A7 probing in an identical manner. For competition assays, *N*. *gonorrhoeae* or *E*. *coli* were applied to nitrocellulose and probed with S100A7-HRP as described, with simultaneous addition of 10-fold molar excess of either mouse S100A7 or unlabeled human S100A7 or S100A12, and signal developed as described above.

### Assay of zinc internalization

Gonococcal cultures were grown as described in Zn-restricted rich medium supplemented with S100A7 and 1 mM IPTG where needed. After 6 hours, cultures were harvested by centrifugation at 3750 RPM for 10 minutes. The pellet was washed with 5 mL cold, Chelex-treated PBS containing 1 mM EDTA. Cultures were then centrifuged and washed again for a total of two washes. This sample was added to a metal-free conical tube with 1 mL set aside for protein quantification via BCA assay. Remaining sample was centrifuged again as described and the pellet was stored overnight at -20°C. After overnight incubation, cell pellets were dissolved in a minimal volume of trace metal grade (67–70%) nitric acid and heated to 95°C for 2 hours, then cooled at room temperature overnight. The resulting samples were diluted to 5% nitric acid by addition of Chelex-treated dH_2_O and submitted for ICP-OES analysis using an Agilent 5110 ICP-OES instrument to detect trace metals compared to a standard curve ranging from 5 to 4000 ppb generated from serial dilutions of a 10 μg/mL multi-element trace metal standard from Inorganic Ventures.

### Accession numbers

**Table:** 

**Accession No.**	**Organism**	**Source**
WP_003706713.1	*Neisseria gonorrhoeae*	NCBI
WP_003697511.1	*Neisseria gonorrhoeae*	NCBI
WP_003692255.1	*Neisseria gonorrhoeae*	NCBI
AAW89864.2	*Neisseria gonorrhoeae*	NCBI
WP_014580101.1	*Neisseria gonorrhoeae*	NCBI
WP_020996886.1	*Neisseria gonorrhoeae*	NCBI
CBX21481.1	*Neisseria lactamica*	NCBI
WP_115118822.1	*Neisseria lactamica*	NCBI
WP_002221107.1	*Neisseria meningitidis*	NCBI
ADY95807.1	*Neisseria meningitidis*	NCBI
WP_045750123.1	*Neisseria lactamica*	NCBI
ADO31914.1	*Neisseria meningitidis*	NCBI
CAM10219.1	*Neisseria meningitidis*	NCBI
EEZ75392.1	*Neisseria lactamica*	NCBI
WP_118816248.1	*Neisseria lactamica*	NCBI
WP_118815216.1	*Neisseria lactamica*	NCBI
WP_029609171.1	*Neisseria lactamica*	NCBI
WP_012221561.1	*Neisseria meningitidis*	NCBI
WP_002244095.1	*Multispecies Neisseriaceae*	NCBI
WP_021437386.1	*Multispecies Neisseriaceae*	NCBI
WP_101065272.1	*Multispecies Neisseriaceae*	NCBI
WP_002248033.1	*Neisseria meningitidis*	NCBI
WP_107970711.1	*Neisseria elongata*	NCBI
WP_096109175.1	*Neisseria lactamica*	NCBI
WP_014574069.1	*Neisseria meningitidis*	NCBI
WP_107854563.1	*Neisseria elongata*	NCBI
WP_107919736.1	*Neisseria elongata*	NCBI
WP_114921675.1	*Neisseria elongata*	NCBI
WP_036469946.1	*Neisseria lactamica*	NCBI
WP_049248486.1	*Neisseria elongata*	NCBI
WP_118898890.1	*Neisseria lactamica*	NCBI
WP_118780086.1	*Neisseria lactamica*	NCBI
WP_003774512.1	*Neisseria elongata*	NCBI
WP_002227407.1	*Neisseria meningitidis*	NCBI
CBN87515.1	*Neisseria lactamica*	NCBI
WP_107992588.1	*Neisseria elongata*	NCBI
WP_114935204.1	*Neisseria lactamica*	NCBI
WP_118819066.1	*Neisseria lactamica*	NCBI
WP_118778219.1	*Neisseria lactamica*	NCBI
WP_115177755.1	*Neisseria lactamica*	NCBI
CAM08367.1	*Neisseria meningitidis*	NCBI
WP_118810395.1	*Neisseria lactamica*	NCBI
WP_118783660.1	*Neisseria lactamica*	NCBI
WP_118786886.1	*Neisseria lactamica*	NCBI
WP_042508227.1	*Neisseria lactamica*	NCBI
WP_118782813.1	*Neisseria lactamica*	NCBI
WP_118846593.1	*Neisseria lactamica*	NCBI
WP_074898586.1	*Neisseria elongata*	NCBI
WP_107884967.1	*Neisseria elongata*	NCBI
WP_002246893.1	*Neisseria meningitidis*	NCBI

## Supporting information

S1 Fig Zn KO S100A7 does not bind zinc. Isothermal titration calorimetry was used to characterize the binding of zinc by wild type S100A7 (blue trace) and the mutant (red trace) designed to have substantially reduced affinity for zinc (S100A7 Zn KO). A clear binding isotherm is observed for the wild type protein (blue trace) and no heat change for the mutant (red trace). These results indicate the zinc affinity of wild type S100A7 (blue trace) is in the nM range, whereas S100A7 Zn KO does not bind zinc with any appreciable affinity.
(TIF)

S2 Fig Integral western blot corresponding to data presented in Fig 3. As our research utilizes a novel α-TdfJ antibody, we show here an uncropped western blot utilized in the generation of Fig 3 of this manuscript. This figure is intended to demonstrate that this antibody is specific for TdfJ and provides little background signal, making it appropriate for the applications demonstrated herein. The signals shown represent TdfJ at the correct MW: 86 kDa. Primary dilution used in this blot was 1:200 in Tris-buffered saline, and secondary antibodies were α-guinea pig conjugated to HRP used at 1:5000.
(TIF)
