# Supplementary figures and images for: The novel interaction between Neisseria gonorrhoeae TdfJ and human S100A7 allows gonococci to subvert host zinc restriction
Source: PLoS Pathog. 2019 Aug 1;15(8):e1007937. doi: 10.1371/journal.ppat.1007937 (PMC6692053; doi:10.1371/journal.ppat.1007937)

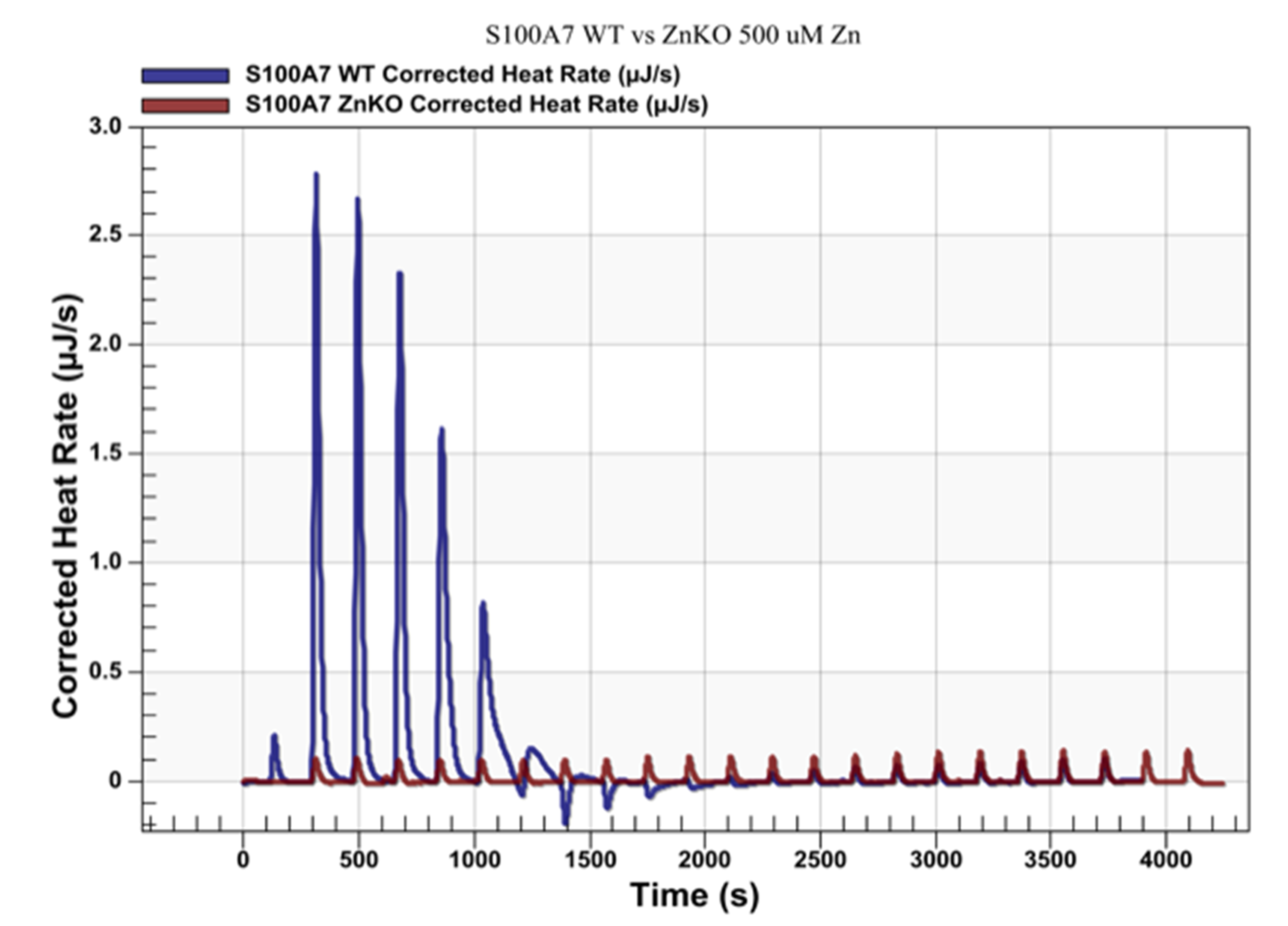

Supplement: S1 Fig — Isothermal titration calorimetry was used to characterize the binding of zinc by wild type S100A7 (blue trace) and the mutant (red trace) designed to have substantially reduced affinity for zinc (S100A7 Zn KO). A clear binding isotherm is observed for the wild type protein (blue trace) and no heat change for the mutant (red trace). These results indicate the zinc affinity of wild type S100A7 (blue trace) is in the nM range, whereas S100A7 Zn KO does not bind zinc with any appreciable affinity. (TIF) [file ppat.1007937.s001.tif]

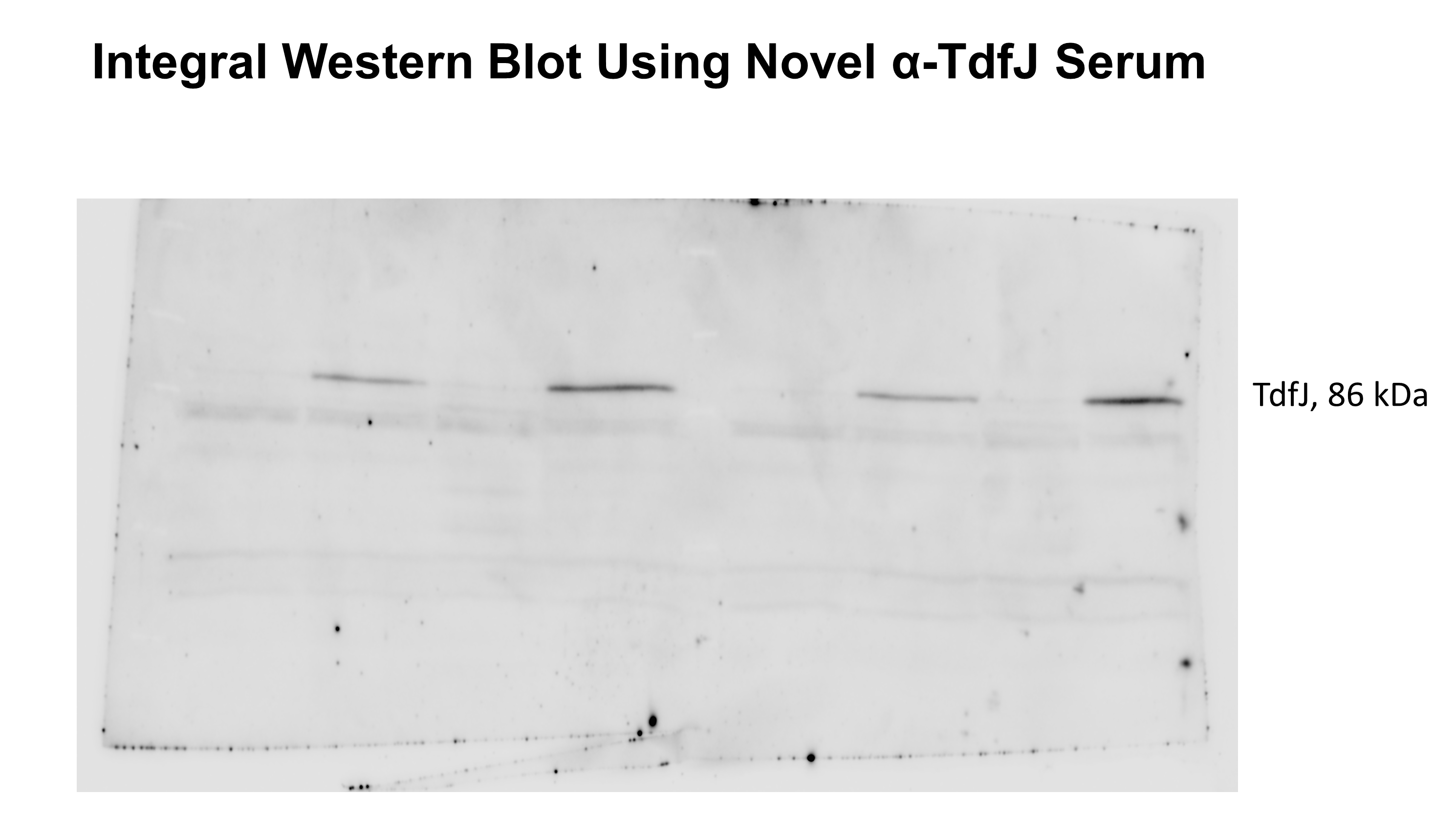

Supplement: S2 Fig — As our research utilizes a novel α-TdfJ antibody, we show here an uncropped western blot utilized in the generation of Fig 3 of this manuscript. This figure is intended to demonstrate that this antibody is specific for TdfJ and provides little background signal, making it appropriate for the applications demonstrated herein. The signals shown represent TdfJ at the correct MW: 86 kDa. Primary dilution used in this blot was 1:200 in Tris-buffered saline, and secondary antibodies were α-guinea pig conjugated to HRP used at 1:5000. (TIF) [file ppat.1007937.s002.tif]
